# Supplementary material for: Finding any Waldo with zero-shot invariant and efficient visual search
Source: Nat Commun. 2018 Sep 13;9:3730. doi: 10.1038/s41467-018-06217-x (PMC6137219; doi:10.1038/s41467-018-06217-x)
Supplement: Supplementary file 1 — Supplementary Information [file 41467_2018_6217_MOESM1_ESM.pdf]

Finding any Waldo with zero-shot invariant and efficient visual search

Zhang et al

1. Supplementary Tables
2. Supplementary Discussion
3. Supplementary Figures
4. Supplementary References

Data and Code Availability. All the raw data and source code are publicly available through the lab's GitHub repository:
https://github.com/kreimanlab/VisualSearchZeroShot

---

1. Supplementary Tables

Random	Pixel	ResNet	AlexNet	VGG16	VGG19
0.17	0.21	0.21	0.22	0.21	0.21

Supplementary Table 1: Category classification performance on 2000 images from 6 selected categories in Experiment 1 using various models based on "low-level" features: pixels, features from first convolution block in ResNet, Alexnet, VGG16, and VGG19 models (Methods). Random indicates performance obtained by selecting one of the 6 categories at random.

2. Supplementary Discussion

Human search for novel objects. All the objects presented in Experiments 1-3 were novel for the IVSN model. Although the human subjects had never seen the exact same objects in Experiments 1 and 2 before, they had extensive prior experience with similar objects from the same categories. Additionally, all human subjects had experience with the Waldo character in Experiment 3. To assess whether human subjects are able to search for objects that they have never encountered before, we conducted an additional experiment using novel objects such as those in **Supplementary Figure 10A** (**Methods**). The structure of the task (**Supplementary Figure 10B**) was similar to the one in Experiment 1 (**Supplementary Figure 1A**), except that the category name was not included. In addition to trials with novel objects, other randomly interleaved trials included the same objects from Experiment 1 (known objects) for direct comparison. To ensure a fair comparison, we matched the difficulty of the task for novel objects and known objects by making the distribution of target - distractor similarity for novel objects close to the corresponding distribution for known objects (**Supplementary Figure 10C**). Humans were able to efficiently find novel objects, with a performance above chance (**Supplementary Figure 10D**, novel objects: 2.42±1.43 fixations, $p<10^{-15}$, t=13, df=2361; known objects: 2.54±1.42 fixations, $p<10^{-15}$, t=12, df=3515). Average performance for novel objects was slightly above performance for known objects (p=0.004, t=2.9, df=5278, two-tailed t-test), but this difference was small and might potentially be attributable to small differences in task difficulty despite our attempts to match the two. We conclude that human subjects are capable of searching for novel objects that they have never encountered before. As expected based on the results in Experiment 1, IVSN was also able to efficiently locate the known and novel objects (**Supplementary Figure 10E**).

Performance on categories not present in ImageNet. The ventral visual cortex part of the model (VGG16 architecture) was pre-trained on 1000 categories from the ImageNet dataset (**Methods**). Although all the images that we used in

Experiments 1 and 2 were different from those in ImageNet, 100 out of 240 of the target categories in Experiment 2 were among the 1000 ImageNet categories. To evaluate whether the IVSN model can generalize to search for target object categories that it has never encountered before, we separately analyzed the 140 target objects from Experiment 2 belonging to categories that are *not* part of ImageNet (**Methods**). There was a small improvement in performance for the 100 images with ImageNet category targets versus the 140 images with novel category targets but this difference was not statistically significant (**Supplementary Figure 5**, p=0.25, two-tailed t-test, t=1.2, df=238). The IVSN model was still able to successfully and efficiently find the target even for categories with zero prior experience.

Image-by-image comparisons. The results presented thus far compared *average* performance between humans and models considering *all* images. We next examined consistency in the responses at the image-by-image level. For a given image, IVSN (e.g., **Figures 3B, 4B, 5B**) and subjects (e.g., **Figures 3C, 4C** and **5C**) go through a sequence of fixations to find the target. We considered different metrics to compare those fixation sequences (**Supplementary Figure 6, Methods**).

We started by considering the total number of fixations required to find the target. First, we evaluated whether subjects would produce a consistent number fixations for the exact same visual search problem in Experiment 1. Unbeknown to subjects, some of the same target and search images were repeated, intermixed in random order, to evaluate the degree of within-subject consistency. The correlation coefficient in the number of fixations required to find the target between the first and repeated instance of the same images ranged from 0.17 to 0.45 (0.31±0.09, **Supplementary Figure 7D**). There was significant variability in each subject's number of fixations under identical task conditions. This definition of within-subject consistency assumes that subjects had no memory over trials; we verified the absence of strong memory effects, which would have been evident as increased values below the diagonal in **Supplementary Figure 7D1**. There was almost no difference between the first and second instances of each image in overall

performance (two-tailed t-tests: Exp1, p=0.96 t=0.06 df=8357; Exp2, p=0.28 t=1.1 df=6011; Exp3, p=0.29 t=1.1 df=1454). Next, we compared whether different subjects required the same number of fixations to find the target. The correlation in the number of fixations between subjects ranged from -0.03 to 0.38 (0.21±0.09, **Supplementary Figure 7D2**). Finally, we compared IVSN to humans and the correlation in the number of fixations ranged from -0.05 to 0.12 (0.03±0.05, **Supplementary Figure 7D3**). Thus, even when the overall performance of IVSN and humans were similar (**Figure 3E**), there were many images that were easy for humans and hard for the model, and vice versa (e.g., **Supplementary Figure 7A**). Subjects were slightly more consistent with themselves than with other subjects, and the between-subject consistency was slightly higher than the consistency with IVSN. These conclusions also extend to Experiments 2 and 3 (**Supplementary Figure 7**).

The number of fixations provides a summary of the efficacy of visual search but does not capture the detailed spatiotemporal sequence of eye movements (**Supplementary Figure 6**). We used the scanpath similarity score[1], to compare two fixation sequences. This metric, derived from comparisons of DNA sequences, captures the spatial distance between saccades in two sequences and their temporal evolution. The similarity score ranges from 0 (maximally different) to 1 (identical sequences). We evaluated scanpath similarity scores within subjects, between subjects and between IVSN and subjects (**Figure 6**). For a fixation sequence of length *x*, we compared the first *x* fixations for all images that had at least *x* fixations. Within-subject comparisons yielded slightly more similar sequences than between-subject comparisons in all 3 experiments ($p<10^{-9}$). The between-subject scanpath similarity scores, in turn, were higher than the IVSN-human similarity scores for all 3 experiments. The IVSN-human similarity scores were higher than the human-chance similarity scores for all 3 experiments. Similar conclusions were reached when comparing all sequences irrespective of their length (**Supplementary Figure 8**), except that the average scanpath similarity score for IVSN-model comparisons was not statistically significant in Experiment 3. In sum, IVSN captured human eye

movement behavior at the image-by-image level in terms of the number of fixations and the spatiotemporal pattern of fixations.

Other ventral visual cortex architectures. We used the VGG16 architecture as an approximation to ventral visual cortex to extract visual features from the target and search images in IVSN (**Figure 2**). There are multiple alternative, yet conceptually similar, deep convolutional architectures including AlexNet[2], ResNet[3] and FastRCNN[4]. In **Supplementary Figure 14**, we report results obtained by replacing the VGG16 visual cortex part of the model by one of those other alternative architectures creating $IVSN_{AlexNet}$, $IVSN_{ResNet}$, and $IVSN_{FastRCNN}$ (**Methods**). All of these models were above chance in all the experiments ($p<0.006$). Overall, the performance of these alternative architectures was similar to that of IVSN but some of them yielded a statistically significant difference with IVSN: $IVSN_{Alexnet}$: $p<0.01$ in Experiment 1; $IVSN_{ResNet}$: $p<10^{-7}$ in Experiment 2 (**Supplementary Figure 14**).

Overt versus covert attention. The IVSN model is agnostic as to whether those attention changes are manifested through overt attention (moving the eyes) or covertly (without moving the eyes). Covert attention changes are harder to quantify at the behavioral level. In the experiments presented here, subjects were instructed to move their eyes to find the target as rapidly as possible. No feedback was provided during the experiment and no punishment was introduced for active exploration via eye movements. The objective was to encourage natural visual search behavior, and avoid alternative strategies such as fixating on the center, and covertly shifting attention until the target was located. The average eye movement reaction times were quite fast (**Supplementary Figure 2**) and were consistent with previous work (e.g.,[5]). While we cannot exclude the possibility that there were covert attention shifts in between saccades, there are only a few tens of milliseconds between the first saccade times (**Figures 3D**, **4D**, **5D**) and the latencies that characterize the visually selective responses along the ventral visual cortex (e.g.,[6]), which does not leave much time for extensive processing or multiple attention shifts.

Visual search in target identical trials. A large body of visual search studies has focused on finding identical matches to a target (e.g.,[5,7,8]). Visual search in the natural world, and most applications of visual search, rarely have the luxury of dealing with identical target search. As expected, performance in such target-identical trials is better than in trials where the target changes shape, both for human subjects as well as for the IVSN model (**Supplementary Figure 3**, **S9C**). Furthermore, even the structure and instructions in the task can have an impact on the results. For example, subjects showed higher performance when all the target-identical trials were blocked (**Supplementary Figure 9D**). Enhanced performance in blocked identical trials may explain why the overall performance in Experiment 1 was slightly lower than in the study of reference[5]. Of course, there are no blocks of target-identical trials in real world visual search and therefore the mixed conditions of Experiments 1-3 better reflect natural search behavior. These results emphasize the need to use randomized trials and transformed versions of the target object to study real world visual search.

Matching bottom-up and top-down weights. The results show that the features learned in an independent object labeling task (training VGG16 via back-propagation using the dataset in ImageNet), can be useful not only in a bottom-up fashion for visual recognition, but also in a top-down fashion to guide feature-based attention changes during visual search. The model assumes that the same bottom-up features are used in a top-down fashion during visual search, i.e., that the top-down weights perfectly match the bottom-up weights. There are biologically-plausible models that are capable of generating top-down weights that follow their bottom-up counterparts[9]. Yet, it remains unclear whether bottom-up synaptic weights are directly matched by top-down synaptic weights in cortex and this assumption will require further evaluation through behavioral and physiological experiments.

Future directions for enhancements to the model. Even when IVSN may approximate human search behavior, the model may not be searching in the same way that humans do. There are several important components of visual search that were simplified in the current model but play an important role in real world visual search, and which may contribute to the enhanced between-subject consistency compared to model-subject consistency.

(i) *Eccentricity dependence*. Human visual acuity drops rapidly from the fovea to the periphery and therefore acuity changes with each saccade. In contrast, the model has perfect acuity through the entire image. Future instantiations of the model should incorporate eccentricity dependence. Combined with potential distance-dependent costs for making saccades (**Supplementary Figure 11G-I**), such eccentricity-dependent acuity may play an important role in biasing the attention map and hence directing saccades.

(ii) *Target recognition*. Once a saccade is made, it is important to decide whether the target is present or not. In the default IVSN, we did not model this recognition component; instead, we used an "oracle" system to decide whether the target was found (the same oracle was used throughout for the human data for fair comparison, except in **Supplementary Figure 12**). As a proof-of-principle demonstration, we implemented a recognition step for each fixation in **Supplementary Figure 11A-C**. This $IVSN_{recognition}$ model performed well in Experiment 1, but slightly less well in Experiments 2 and 3 where there is significant clutter. There has been extensive work on invariant visual recognition systems that could be incorporated into IVSN to decide whether the target is present or not[2,3,10,11]. It should be noted that humans also make recognition mistakes. Examples of such mistakes are shown in **Figures 4C** and **5C** where subjects moved their eyes to the target location, yet did not click the mouse to indicate that they had found the target, **Supplementary Figure 12**). It is conceivable that in some of those cases, subjects did consciously recognize the target but wanted to be certain

and thus decided to further explore the image; this should not be described as a recognition failure but rather a decision-making failure. However, it is more likely, particularly in Experiment 3, that in most of those cases subjects fixated on the target, yet failed to recognize it.

(iii) *Memory*. The default IVSN model (and all null models) had infinite inhibition-of-return, that is, they never went back to the same location. In contrast, humans revisit the same location even if the target is not there (e.g., **Figure 4C**, **5C**, **3E**, **4E**, **5E**,[12,13]). We implemented a memory function in $IVSN_{fIOR}$ by fitting human behavioral data such that the model could probabilistically go back to previous locations (**Supplementary Figure 11D-F**). The combination of (ii) and (iii) is probably important and relevant. Under the oracle system (perfect recognition), there is no incentive in revisiting previous locations. However, when considering an imperfect recognition machinery that can make mistakes, an imperfect memory may be useful to endow the model with the possibility of revisiting a given location where the target may have been present. Even though the recognition machinery in the models considered here is deterministic, the exact fixation center could be different when revisiting a location and this could lead to correct recognition.

(iv) *Learning*. There is no training in the models presented in this study. The ventral visual cortex was extensively pre-trained for visual object recognition but that training was not part of this study. IVSN capitalizes on those weights learned for visual recognition through a series of operations imposed to do visual search. Those operations could be learnt. The visual system could learn how to generate a sequence of fixations, including the interaction of the different bottom-up, top-down, memory and recognition components, the winner-take-all mechanism, inhibition-of-return, saccade size constraints, decisions about whether the target is present or not, etc.. An elegant idea on how learning could be implemented was presented in ref.[14] where the authors proposed an architecture that can learn to generate eye movements via reinforcement

learning with a system that is rewarded when the target is found. The generation of the attention map in the IVSN model is end-to-end trainable. IVSN can be improved by training or fine-tuning via reinforcement learning for various search tasks depending on the applications.

(v) *Information from previous saccades*. Previous saccades are incorporated through the inhibition-of-return mechanism (to avoid visiting previous locations) and through the saccade distance constraint (precluding from making very large saccades). Beyond these two elements, saccades are considered to be independent. However, a complete model should incorporate inter-dependences across saccades by using visual information obtained during previous fixations to guide the next saccade. This is particularly relevant in combination with (i). IVSN has access to a complete high resolution map of the entire image. However, the human visual system only has high-resolution information in the fovea. Each subsequent fixation provides additional high-resolution information at a different location in the image and this information should be incorporated to better guide the next fixation.

(vi) *Cognitive knowledge about the world*. The images in Experiment 3, and particularly those in Experiment 1, violate basic components of real world images. In real world images (Experiment 2), subjects may capitalize on high-level knowledge about scenes[8,15] including understanding certain statistical correlations in object positions (e.g., it is highly unlikely that the car keys would be glued to the ceiling), basic properties of the physical world (an object needs support and therefore keys are more likely to be found on top a desk or the floor rather than floating in the air), correlations in object sizes (the size of a phone in the image may set an expectation for the size of the keys), etc. Such knowledge can place significant constraints on the visual search problem, leading to adequately skipping search over large parts of an image. None of this high-level knowledge is incorporated into the IVSN model.

***Relationship to object detection and image retrieval in computer vision*.** Traditional template-matching computational algorithms do not perform well in invariant object recognition. In visual search tasks, template-matching shows selectivity to distinguish an identical target from distractors, but fails to robustly find transformed versions of the target. To circumvent this problem, investigators have developed object detection, object localization, and image retrieval approaches which can successfully and robustly localize objects, at the expense of having to extensively train those models with the sought targets and exhaustively scan the image through sliding windows[4]. To localize objects, recent work focuses on deep neural networks requiring a large amount of supervised data, such as bounding boxes or object segmentations[4,16,17]. Typically, these approaches either use a sliding window or propose regions of interest uniformly over a grid, performing feed-forward classification for each region and making decisions about the presence or absence of the target. An analogous strategy is used in image retrieval tasks where a similarity score is computed between a query and each candidate image[18,19]. These heuristic methods are computationally inefficient (in terms of the number of ``fixations" or proposed regions required to find the target), and require extensive class-specific training.

Supplementary Figure 1

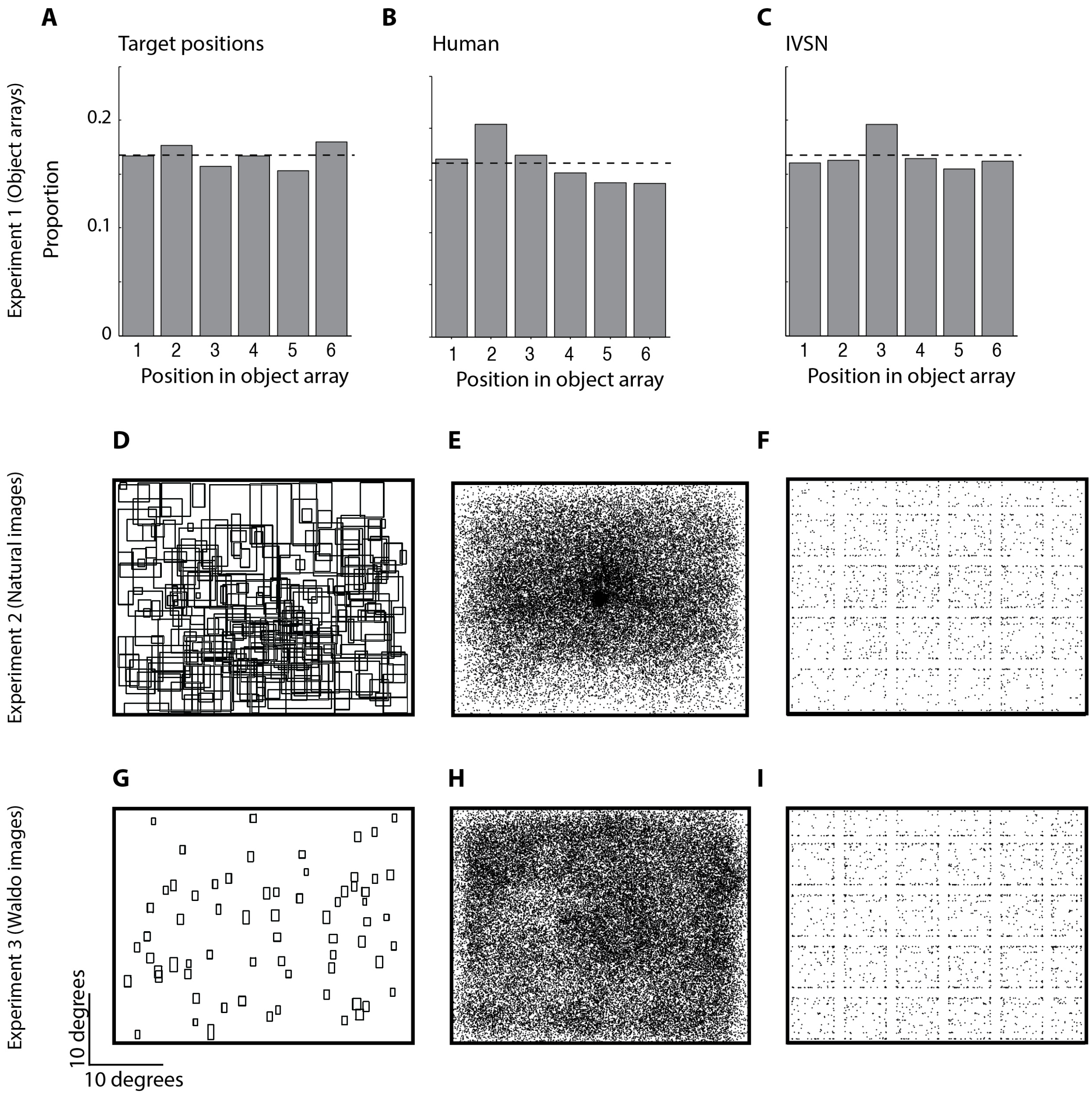

Supplementary Figure 1. Overall distribution of target locations, human fixations and model fixations. **A, D, G**. Distribution of target locations for Experiment 1, 2 and 3, respectively. **B, E, H**. Distribution of human subject fixations for 15 subjects in each experiment (n=31,202, 99,610 and 71,346 fixations for Experiments 1, 2 and 3, respectively). In panel **H**, there is a slightly lower density of fixations in a section in the upper left quadrant; in 13/67 images, this location had text instructions and subjects were instructed to avoid this area. **C, F, I**. Distribution of IVSN fixations. The white spacing between fixations in the model is due to the way in which the large images were cropped in order to feed smaller size image segments into the model (**Methods**).

Supplementary Figure 2A-F

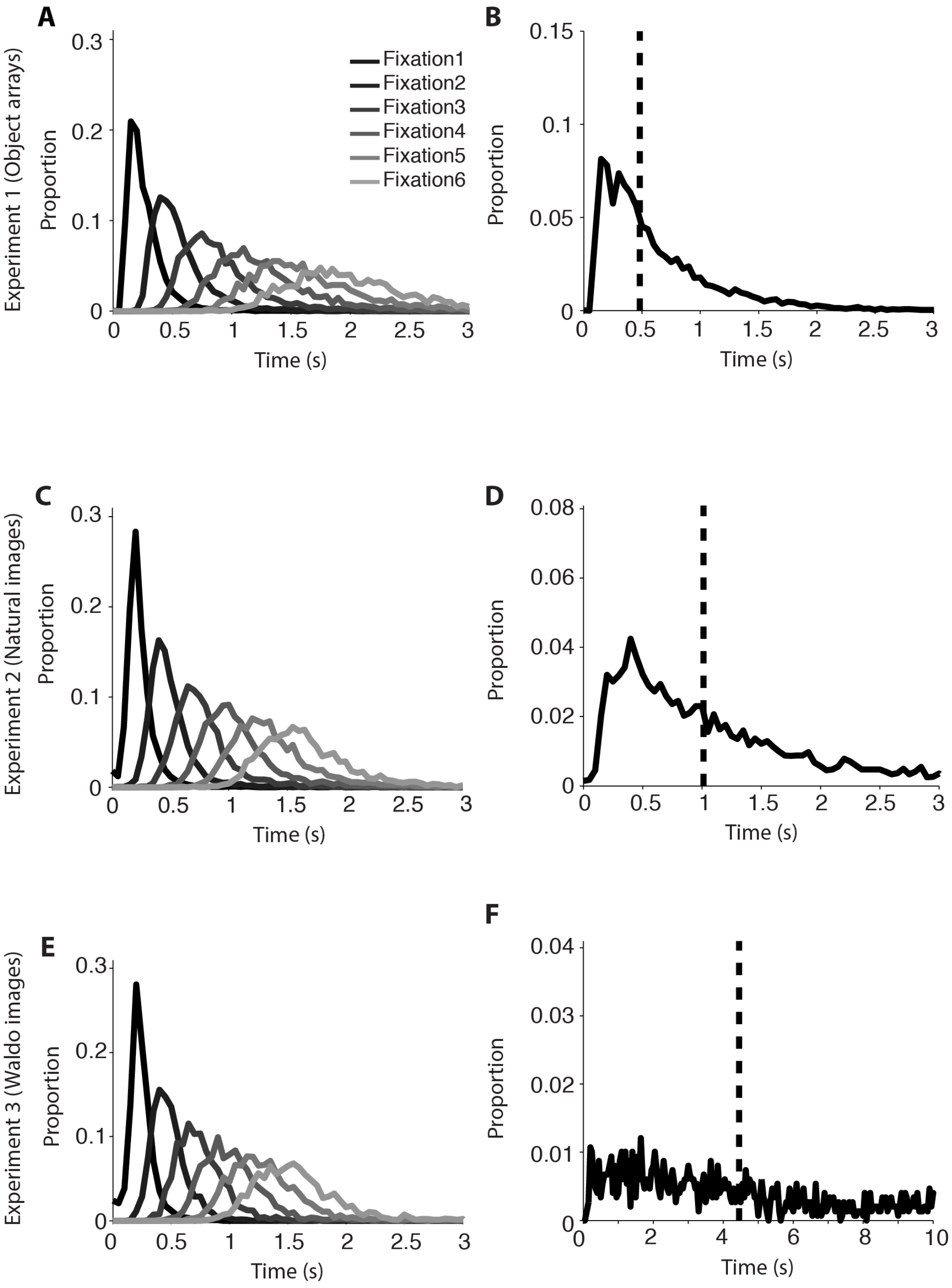

Supplementary Figure 2. Distribution of reaction times and saccade sizes. A, C, E. Distribution of reaction times for the first 6 fixations, across 15 subjects, for Experiments 1, 2 and 3, respectively. Bin size = 50 ms. In Experiments 2 and 3, there were many trials with >6 fixations (**Figure 4E**, **5E**). The distribution for the first fixation is the same as the one shown in **Figures 3D**, **4D**, and **5D** and is reproduced here for completeness. The x-axis was cut at 3 seconds. **B**, **D**, **F**. Distribution of time required to find the target, across 15 subjects. Bin size = 50 ms. The vertical dashed line denotes the median (mean values are reported in the text). There was a significant difference in the time required to find the target among the 3 experiments (one-way ANOVA, $p<10^{-15}$, df=14217, F=3015).

Supplementary Figure 2G-L

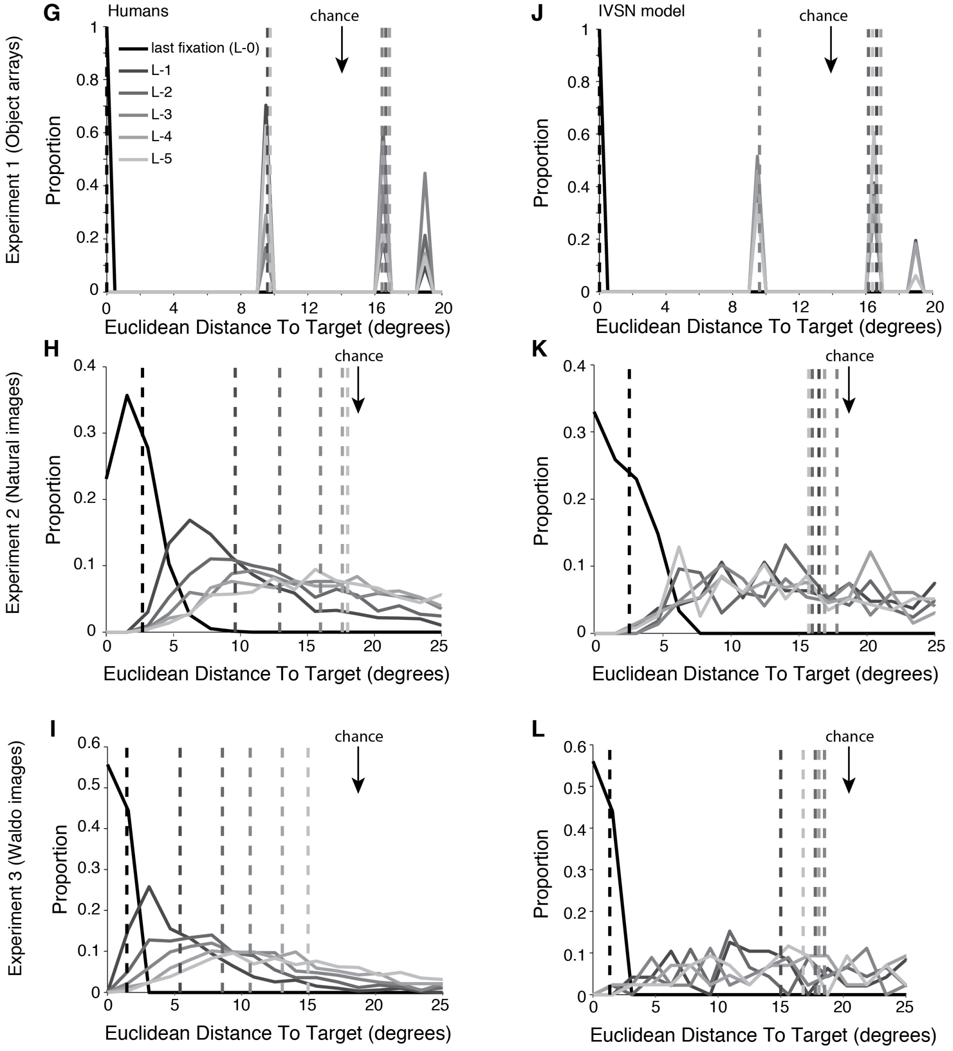

Supplementary Figure 2G-L. Distance to target for the last 6 fixations. Distribution of the distance (in degrees of visual angle) between the fixation location and the target location for the last fixation (L-0), the fixation before last (L-1), etc. for humans (**G-I**) or the IVSN model (**J-L**). The vertical dashed lines denote the average of each distribution. On average, each subsequent fixation brought human subjects closer to the target towards the end, whereas the model was more likely to arrive at the target from a distant location. The arrows indicate the expected distance from a random location to the target. In **H, I, K, L**, given the image dimensions L_w=40 degrees, L_h=32 degrees and d=sqrt(L_w^2+L_h^2), this chance distance is given by:

$$\frac{1}{15}\left(\frac{L_w^3}{L_h^2}+\frac{L_h^3}{L_w^2}+d\left(3-\frac{L_w^2}{L_h^2}-\frac{L_h^2}{L_w^2}\right)+2.5\left(\frac{L_h^2}{L_w}ln\left(\frac{L_w+d}{L_h}\right)+\frac{L_w^2}{L_h}ln\left(\frac{L_h+d}{L_w}\right)\right)\right)$$

Supplementary Figure S3

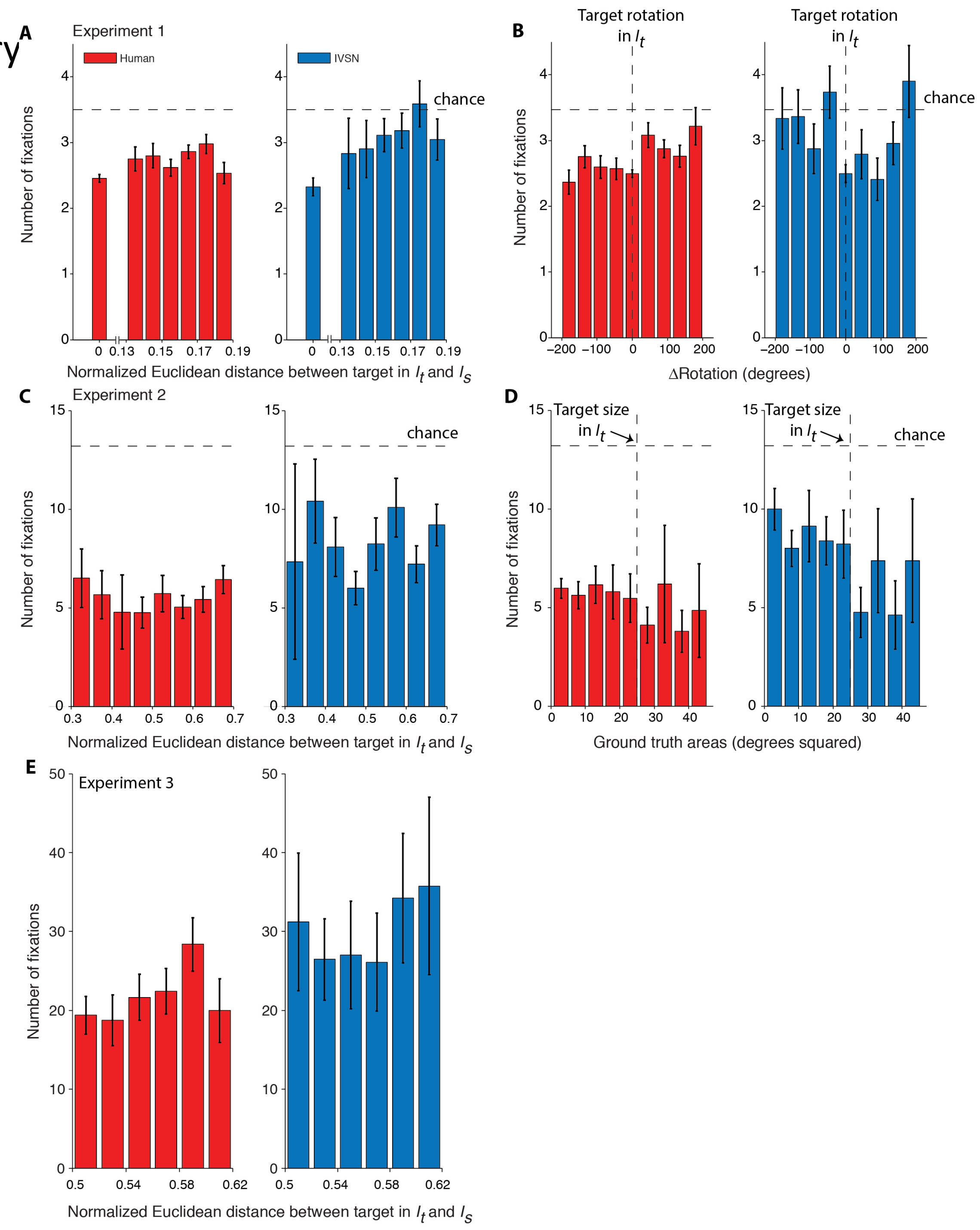

Supplementary Figure 3. Invariance in visual search.
A. Number of fixations required to find the target in Experiment 1 as a function of the distance between the target as rendered in the I_t and I_s images. Distance = 0 corresponds to identical targets (note cut in x-axis). The horizontal dashed line indicates the null chance model. Error bars denote SEM.
B. Number of fixations required to find the target in Experiment 1 as a function of the difference between the rotation of the target object in the target image and in the search image for humans (red) and the IVSN model (blue). The vertical dashed line indicates those trials where the target was shown with the same 2D rotation angle in the I_t and I_s images. The horizontal dashed line indicates the null chance model.
C. Similar to **A** for Experiment 2. There were no distance=0 trials in this experiment.
D. Number of fixations required to find the target in Experiment 2 as a function of the area of the target in the I_s image. The dashed line shows the size of the target object in the I_t image. The horizontal dashed line indicates the null chance model.
E. Similar to **A** for Experiment 3. The null chance model required 58 fixations on average (beyond the y scale).

Supplementary Figure 4A

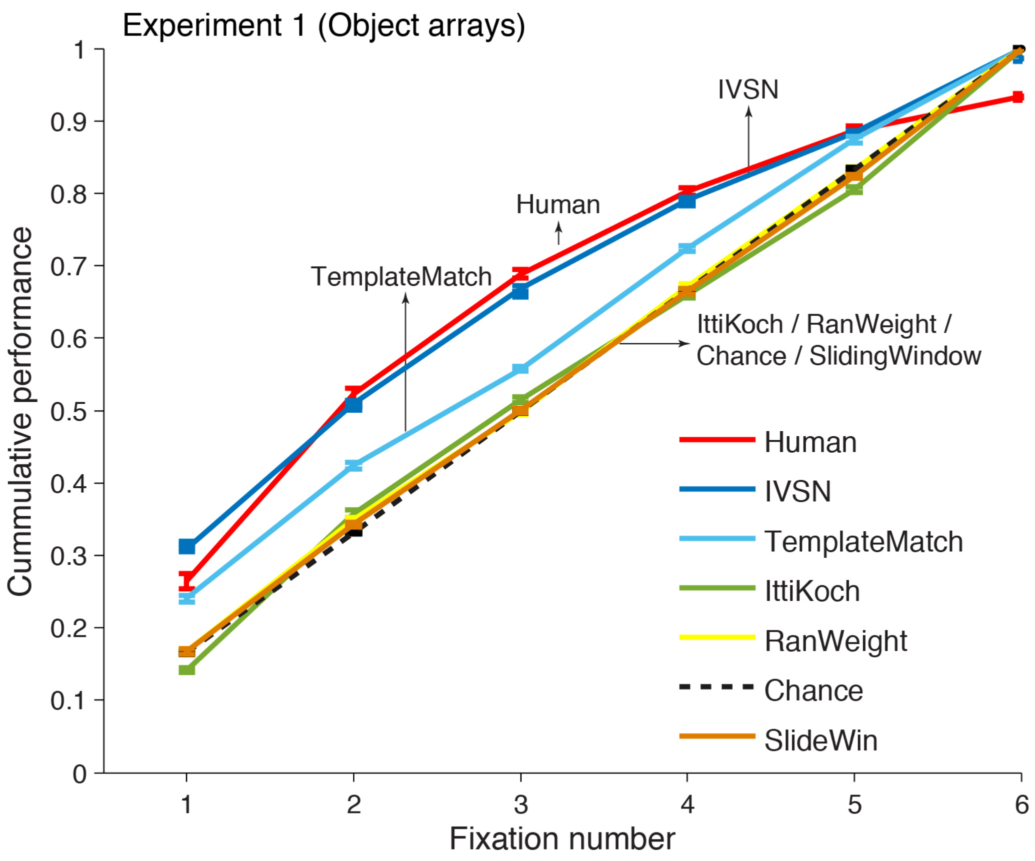

Supplementary Figure 4. Performance comparison with alternative models. The format and conventions are the same as those in **Figures 3E**, **4E**, **5E** in the main text. Error bars denote SEM. See text and Methods for a description of each model. The curves for "Human", IVSN, and Chance are reproduced from **Figures 3E**, **4E** and **5E** for comparison purposes. **A.** Experiment 1 (Object arrays).

All models except for IVSN were statistically different from humans (two-tailed t-test):

TemplateMatching: $p<10^{-9}$,
RanWeight: $p<10^{-15}$
IttiKoch: $p<10^{-15}$
SlideWin: $p<10^{-15}$
Chance: $p<10^{-15}$
IVSN: p=0.03

All models were statistically different from IVSN (p<0.01, two-tailed t-test, df>598).

TemplateMatching: p=0.01
RanWeight: $p<10^{-5}$
IttiKoch: $p<10^{-6}$
SlideWin: $p<10^{-7}$
Chance: $p<10^{-12}$

Supplementary Figure 4B

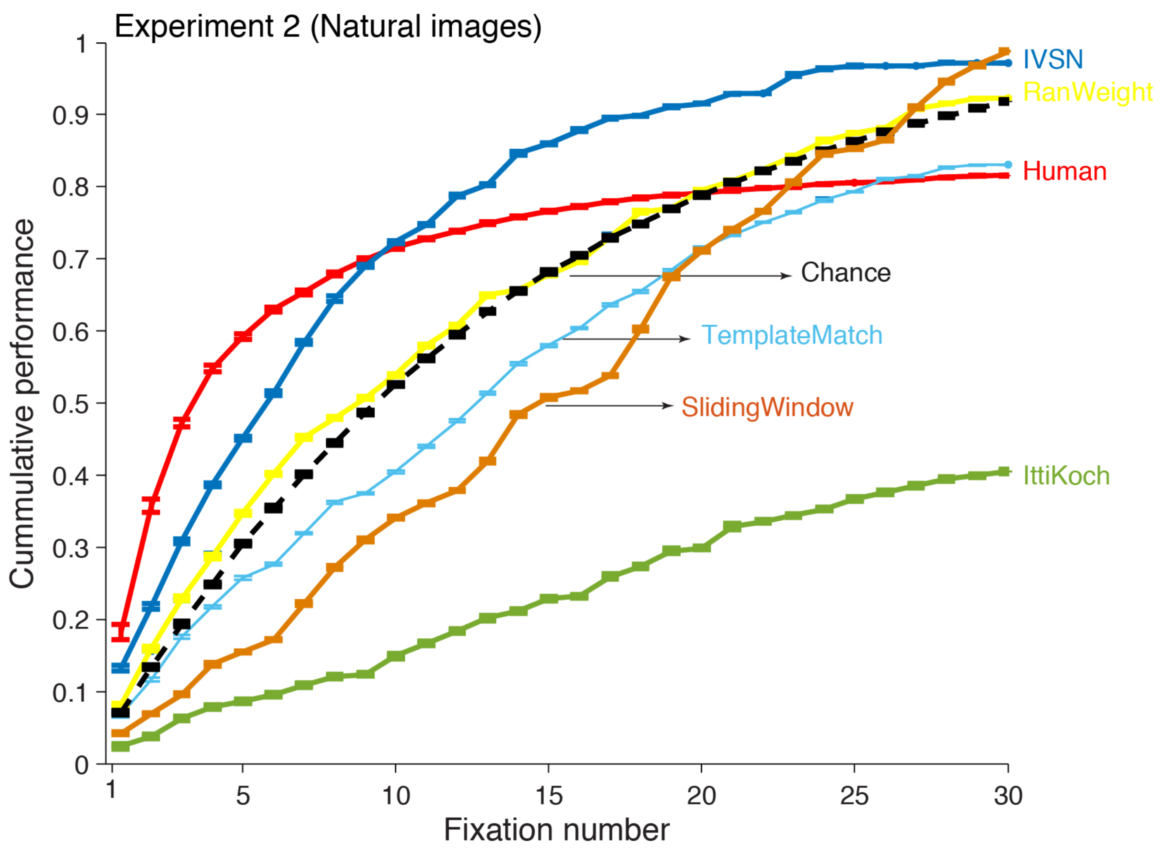

Supplementary Figure 4. Performance comparison with alternative models.
B. Experiment 2 (Natural images).

All models were statistically different from humans (two-tailed t-test):
TemplateMatching: $p<10^{-15}$,
RanWeight: $p<10^{-15}$
IttiKoch: $p<10^{-15}$
SlideWin: $p<10^{-15}$
Chance: $p<10^{-15}$
IVSN: $p<10^{-5}$

All models were statistically different from IVSN (two-tailed t-test):
TemplateMatching: $p<10^{-10}$
RanWeight: $p<10^{-5}$
IttiKoch: $p<10^{-15}$
SlideWin: $p<10^{-15}$
Chance: $p<10^{-15}$

Supplementary Figure 4C

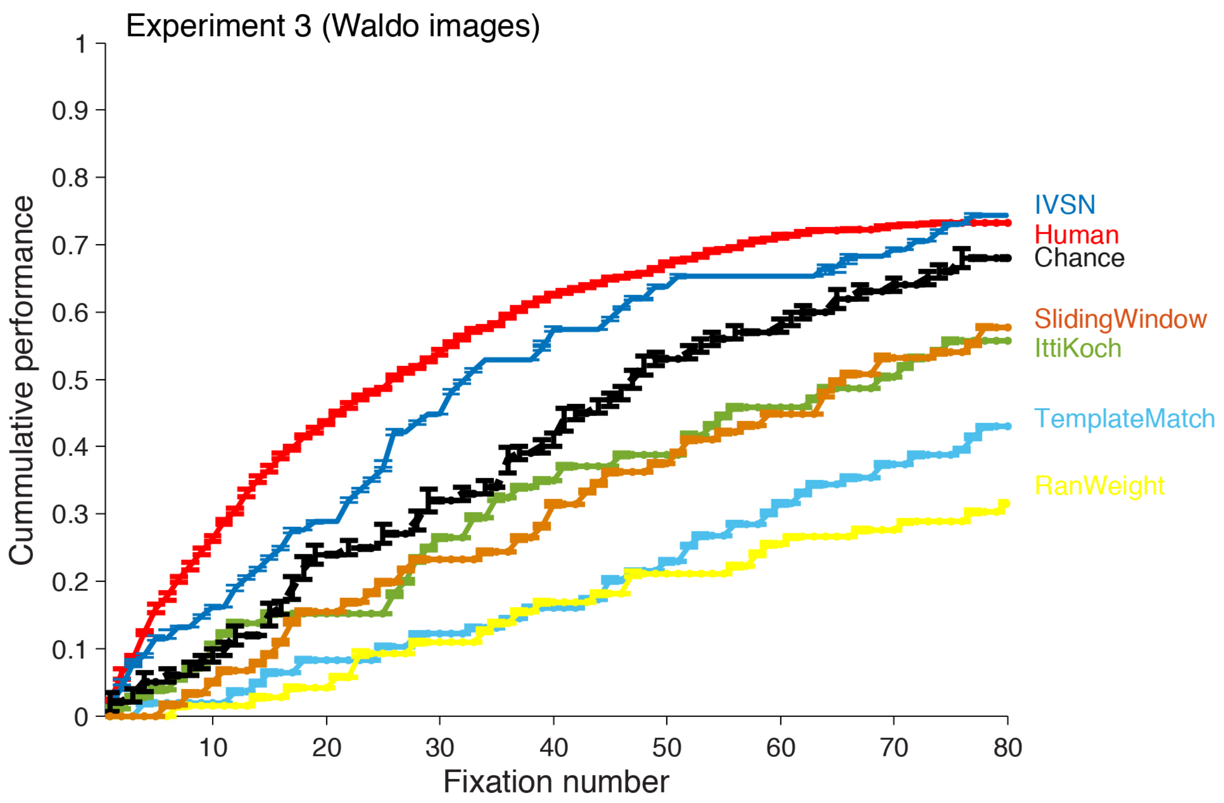

Supplementary Figure 4. Performance comparison with alternative models.
C. Experiment 3 (Waldo images).

All models were statistically different from humans (two-tailed t-test):
TemplateMatching: $p<10^{-13}$,
RanWeight: $p<10^{-8}$
IttiKoch: $p<10^{-6}$
SlideWin: $p<10^{-15}$
Chance: $p<10^{-15}$
IVSN: p=0.001

All models were statistically different from IVSN (two-tailed t-test):
TemplateMatching: p=0.001
RanWeight: $p<10^{-8}$
IttiKoch: p<0.01
SlideWin: $p<10^{-8}$
Chance: $p<10^{-15}$

Supplementary Figure 5

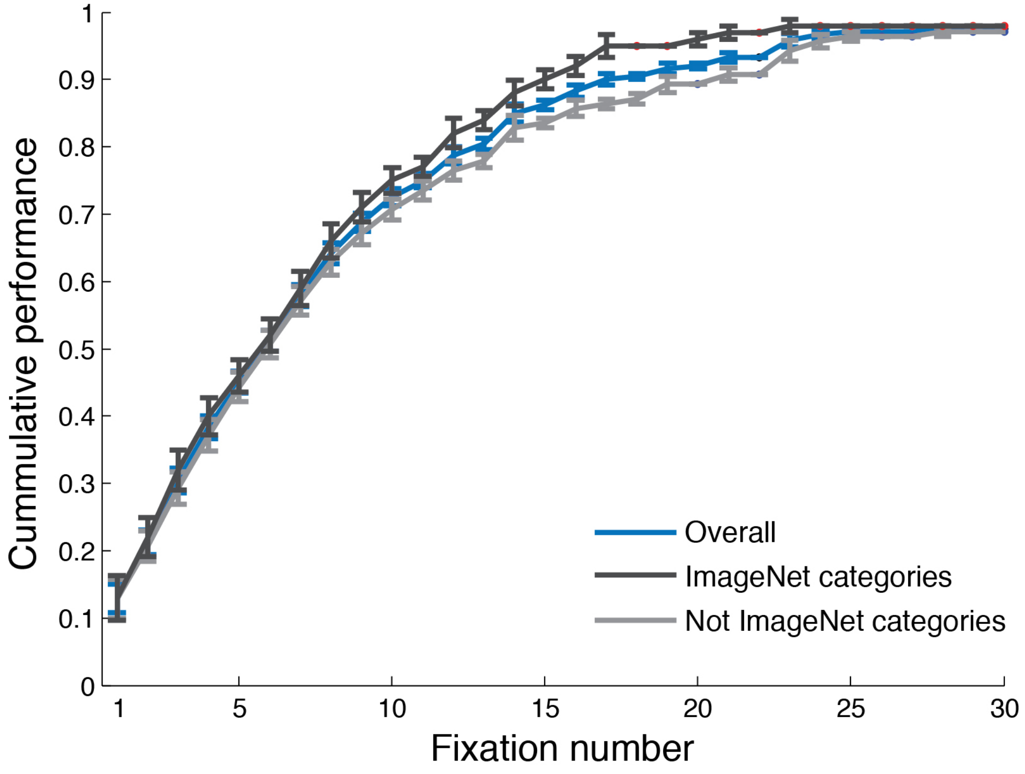

Supplementary Figure 5. Performance for ImageNet categories and non-ImageNet categories in Experiment 2. Following the format in **Figure 4E**, cumulative performance as a function of fixation number for all images (blue, same copied from **Figure 4E**), 100 images with target object categories that were within ImageNet (dark gray) and 140 that did not (light gray). Although performance was slightly higher for target objects in ImageNet categories, there was no significant difference between the number of fixations required to find the target for ImageNet or non-ImageNet images (p=0.25, two-tailed t-test, t=1.2, df=238). Error bars denote SEM.

Supplementary Figure 6

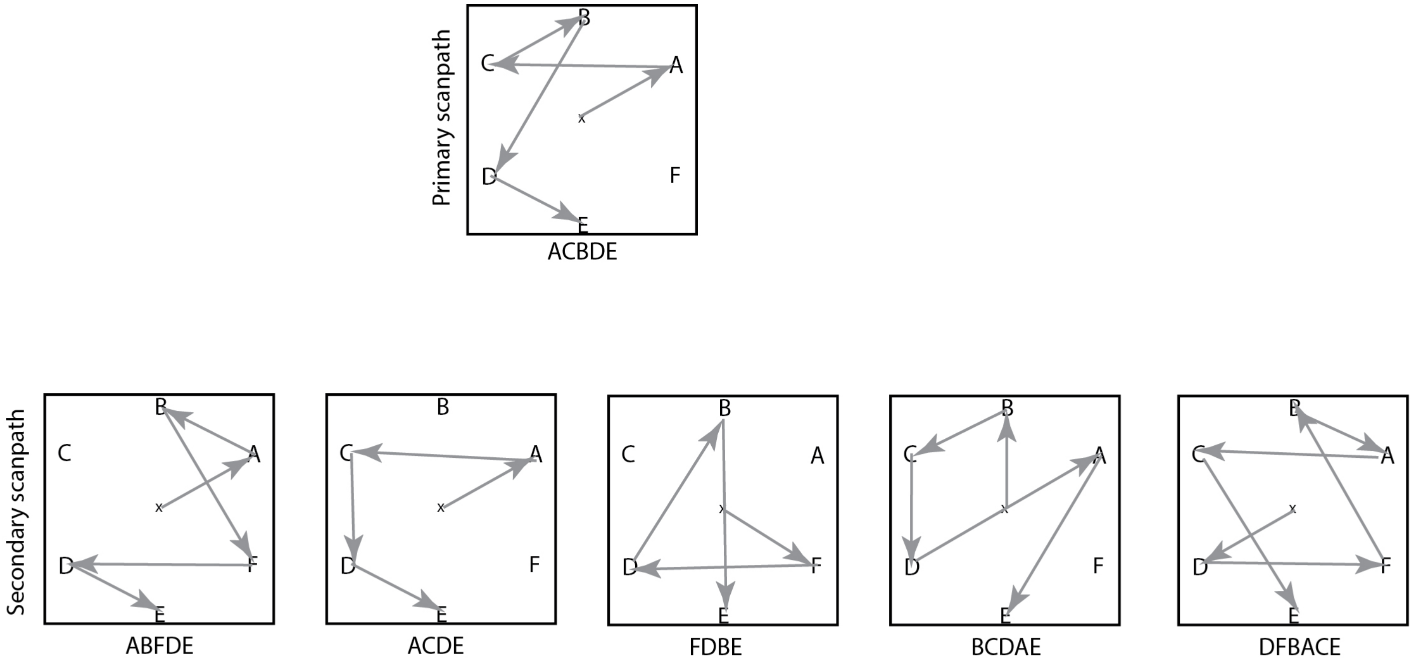

	ABFDE	ACDE	FDBE	BCDAE	DFBACE
Δ(Number of fixations)	0	1	1	0	-1
Scanpath score	0.75	0.75	0.50	0.75	0.40

Supplementary Figure 6. Illustration of image-by-image consistency metrics in fixation patterns. This schematic shows a comparison between a primary scan path (top, sequence = ACBDE) and alternative scan paths (middle) in a search image consisting of 6 objects where the target is at location E. The numbers below each subplot show the difference in the number of fixations and the scan path similarity score for each comparison with the primary scan path (**Methods**).

Supplementary Figure 7A

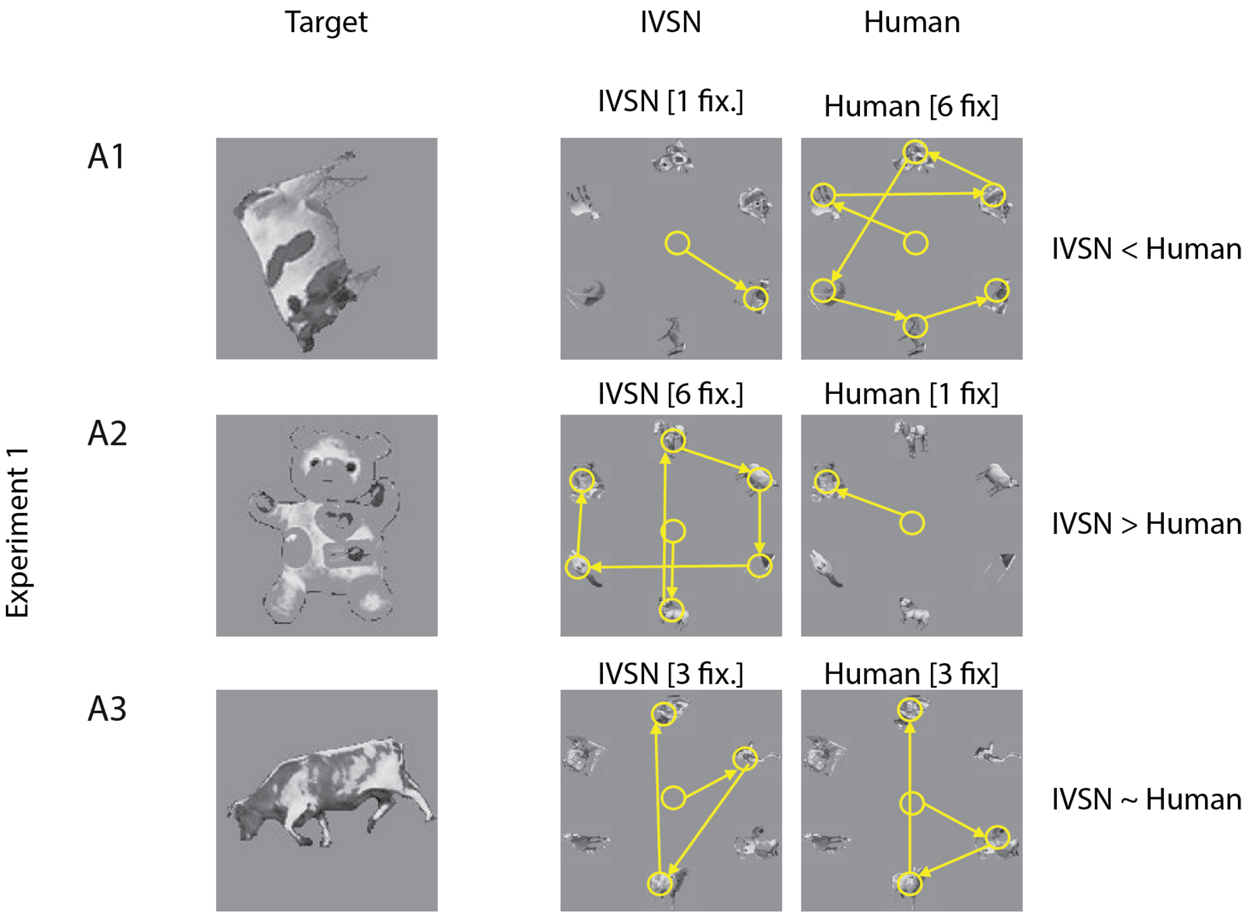

Supplementary Figure 7. Image-by-image comparison of number of fixations required to find the target. A-C. Example trials where the IVSN model found the target faster than humans (**A1, B1, C1**), when humans found the target faster than the IVSN model (**A2, B2, C2**), and trials where humans and the IVSN model were comparable (**A3, B3, C3**) for Experiment 1 (**A**), Experiment 2 (**B**), and Experiment 3 (**C**). The left column shows the target image, columns 2 and 3 show the sequence of fixations for the IVSN model (column 2) and one of the subjects (column 3). The number of fixations required to find the target is shown above each search Image.

Supplementary Figure 7B

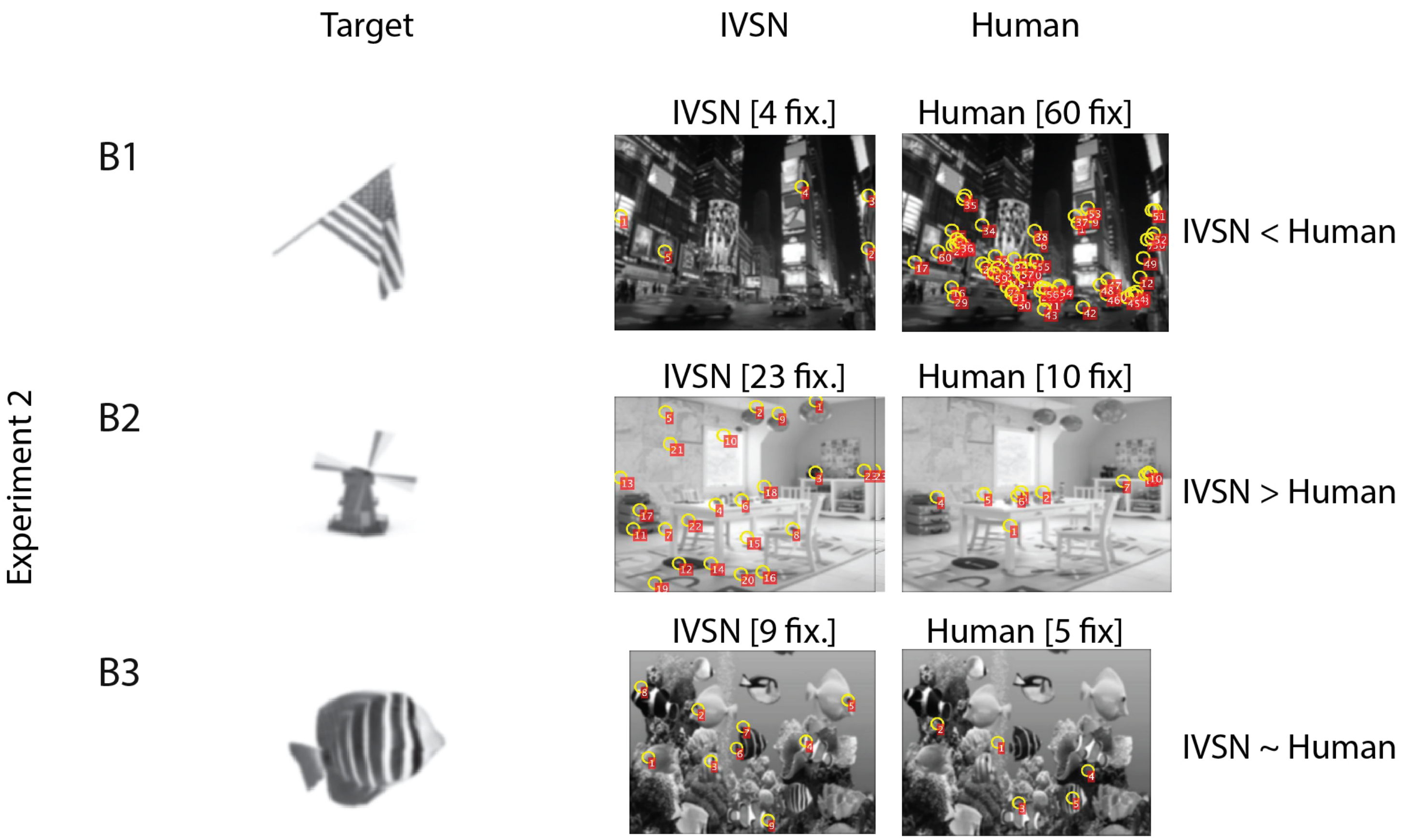

Supplementary Figure 7. Image-by-image comparison of number of fixations required to find the target. See previous panel for legend.

Supplementary Figure 7C

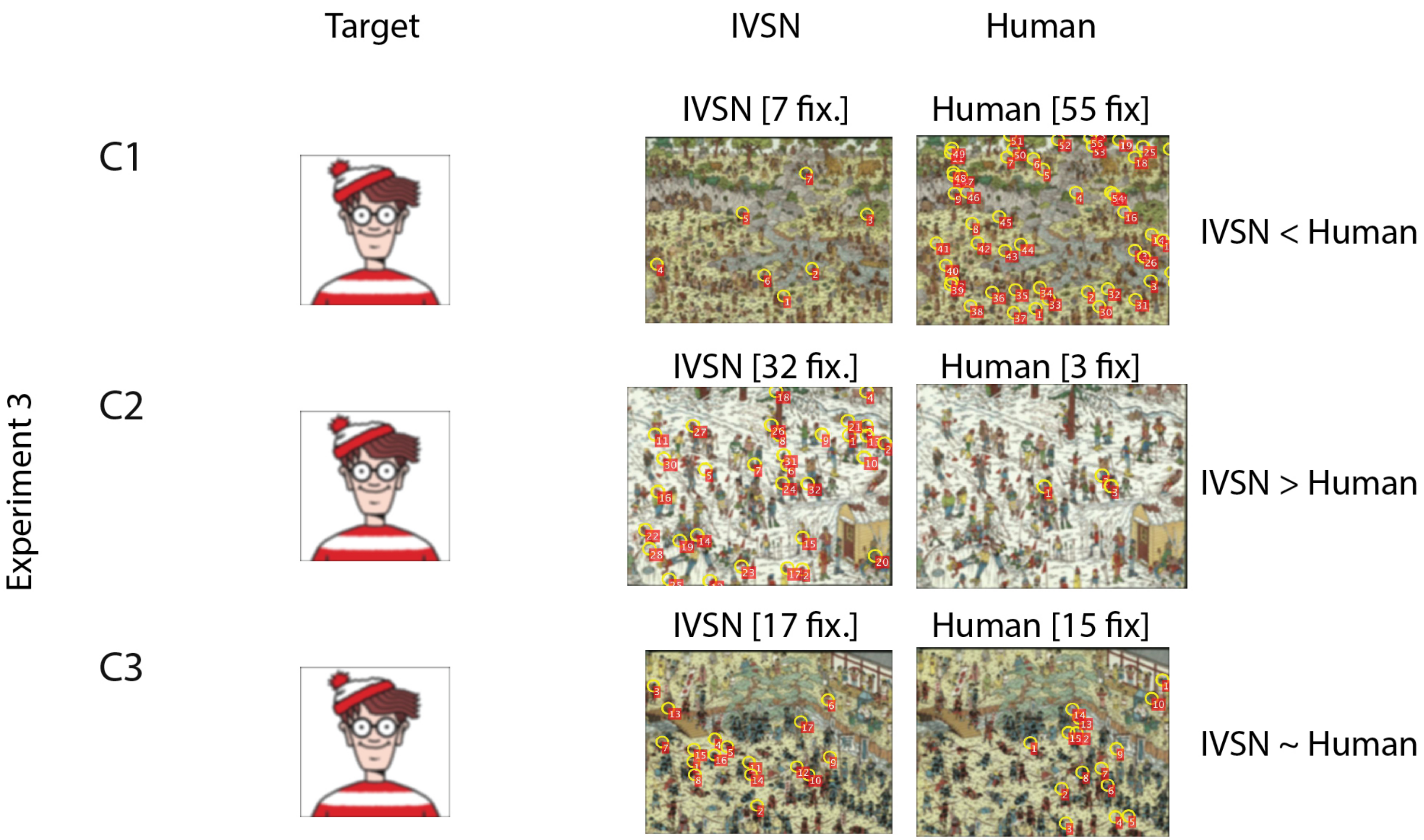

Supplementary Figure 7. Image-by-image comparison of number of fixations required to find the target. See previous panel for legend.

Supplementary Figure 7DEF

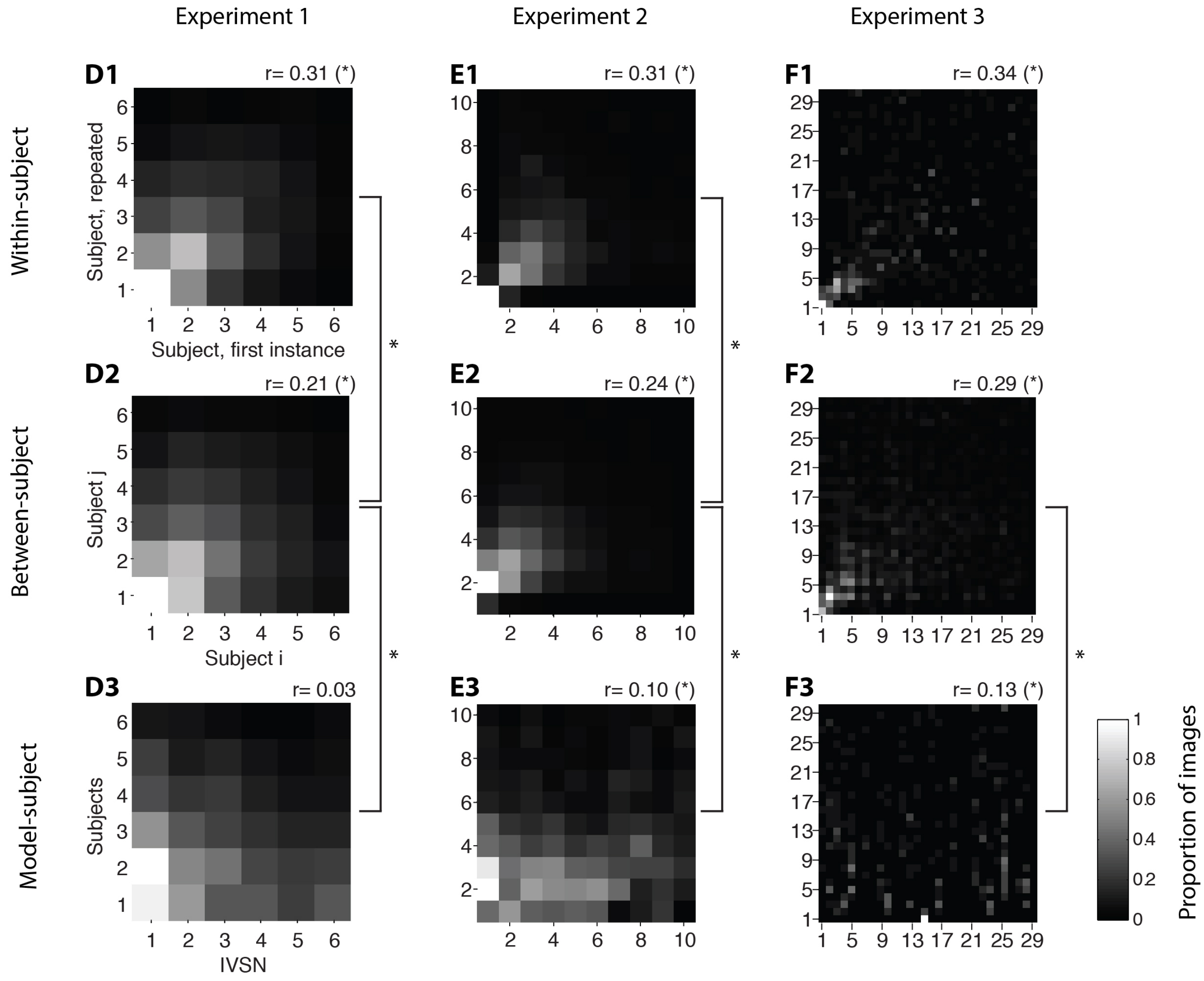

Supplementary Figure 7. Image-by-image comparison of number of fixations required to find the target. D-F. Comparison in the number of fixations required to find the target averaged across subjects for each experiment (columns), within subjects (**D1**, **E1**, **F1**), between subjects (**D2**, **E2**, **F2**) and between subjects and IVSN model (**D3**, **E3**, **F3**). When comparing S1 and S2 (e.g., two subjects), entry (*i,j*) indicates the proportion of images where S1 required *i* fixations and S2 required *j* fixations (see scale bar on bottom right). Presence of entries exclusively along the diagonal would indicate that the behavior of S1 and S2 is identical on an image-by-image basis. Results were averaged across subjects (see **Figures S7G-I** for distribution for individual subjects). Note that the size of the matrices are different for each experiment, reflecting the increasing difficulty from Experiment 1 to 3. The r values show the average of the correlation coefficients computed in **Figures S7G-I** in the subject-by-subject comparisons. An * next to the *r* value indicates that the distribution of *r* values was different from zero (two-tailed t-test, $p<0.01$). An * comparing two matrices indicates that the distributions of *r* values were statistically different (two-tailed t-test, $p<0.01$).

Supplementary Figure 7GHI

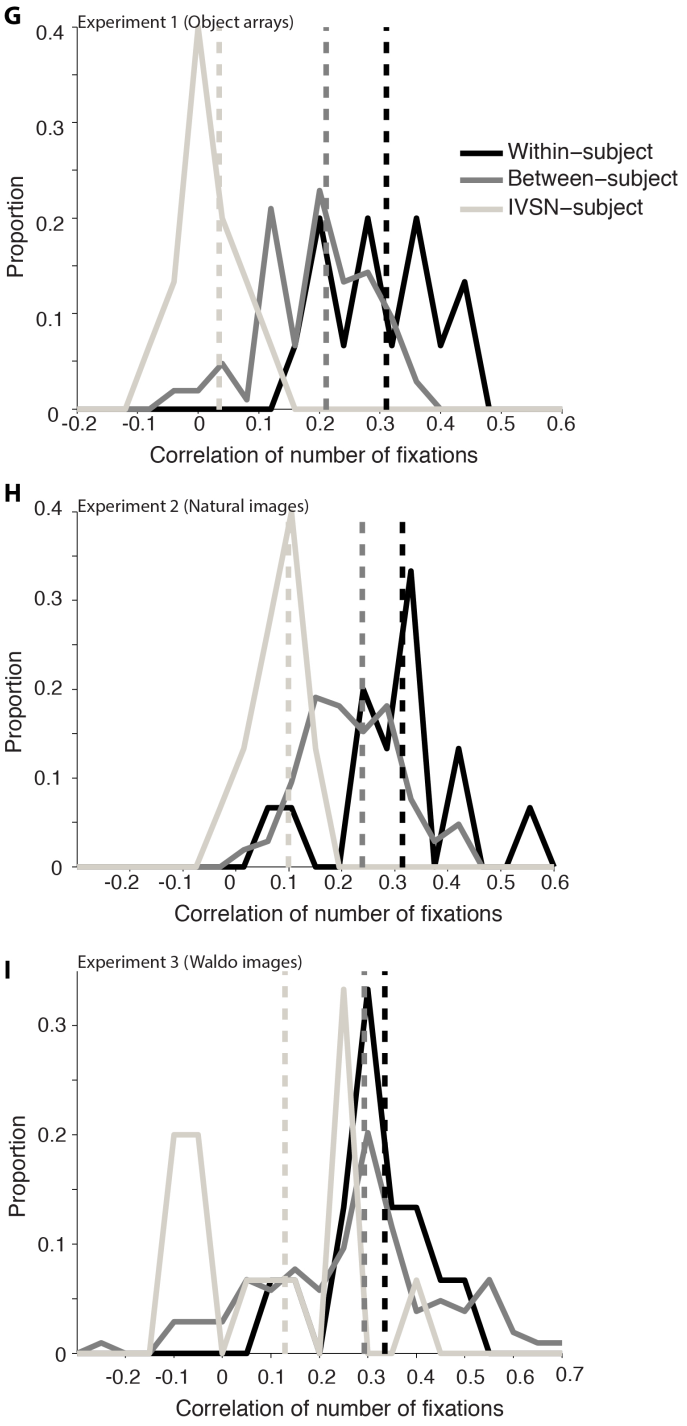

Supplementary Figure 7. Image-by-image comparison of number of fixations required to find the target. Using the same comparison of the number of fixations described for **Figure S7DEF**, this figure shows the distribution of the correlation coefficients on a subject-by-subject basis for Experiment 1 (**G**), Experiment 2 (**H**) and Experiment 3 (**I**). The colors denote the within-subject comparisons (black), between subject comparisons (dark gray), and IVSN-subject comparisons (light gray).

Supplementary Figure 8ABC

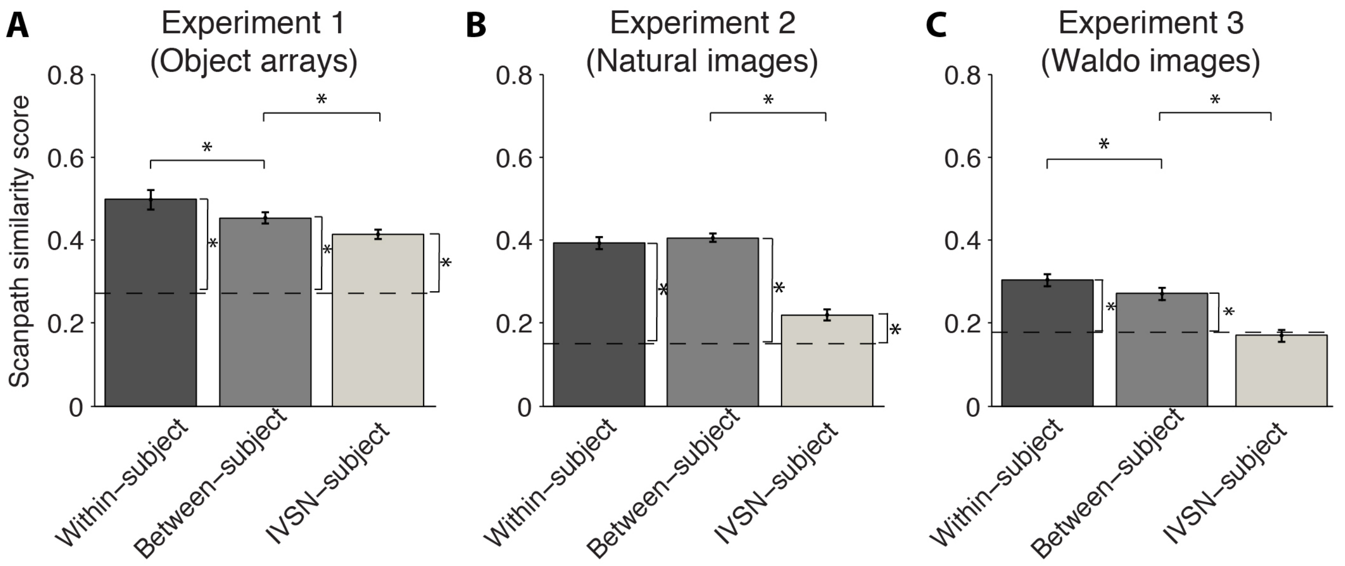

Supplementary Figure 8: Image-by-image consistency in the spatiotemporal pattern of fixation sequences using entire fixation sequences. Scanpath similarity scores (see text and **Methods** for definition) comparing the fixation sequences within subjects (dark gray), between-subjects (medium gray) and between the IVSN model and subjects (light gray) for Experiment 1 (**A**), Experiment 2 (**B**), and Experiment 3 (**C**). The larger the scanpath similarity score, the more similar the fixation sequences are. The dashed line indicates chance performance, obtained by randomly permuting the images. Results shown here are averaged over subjects and subject pairs. The “*” denote statistical significance ($p<0.01$, two-tailed t-test), comparing each result against chance levels (vertical comparisons) and comparing within-subject versus between-subject scores and between-subject versus IVSN-subject scores (horizontal comparisons). Error bars denote SEM.

Supplementary Figure 8DEF

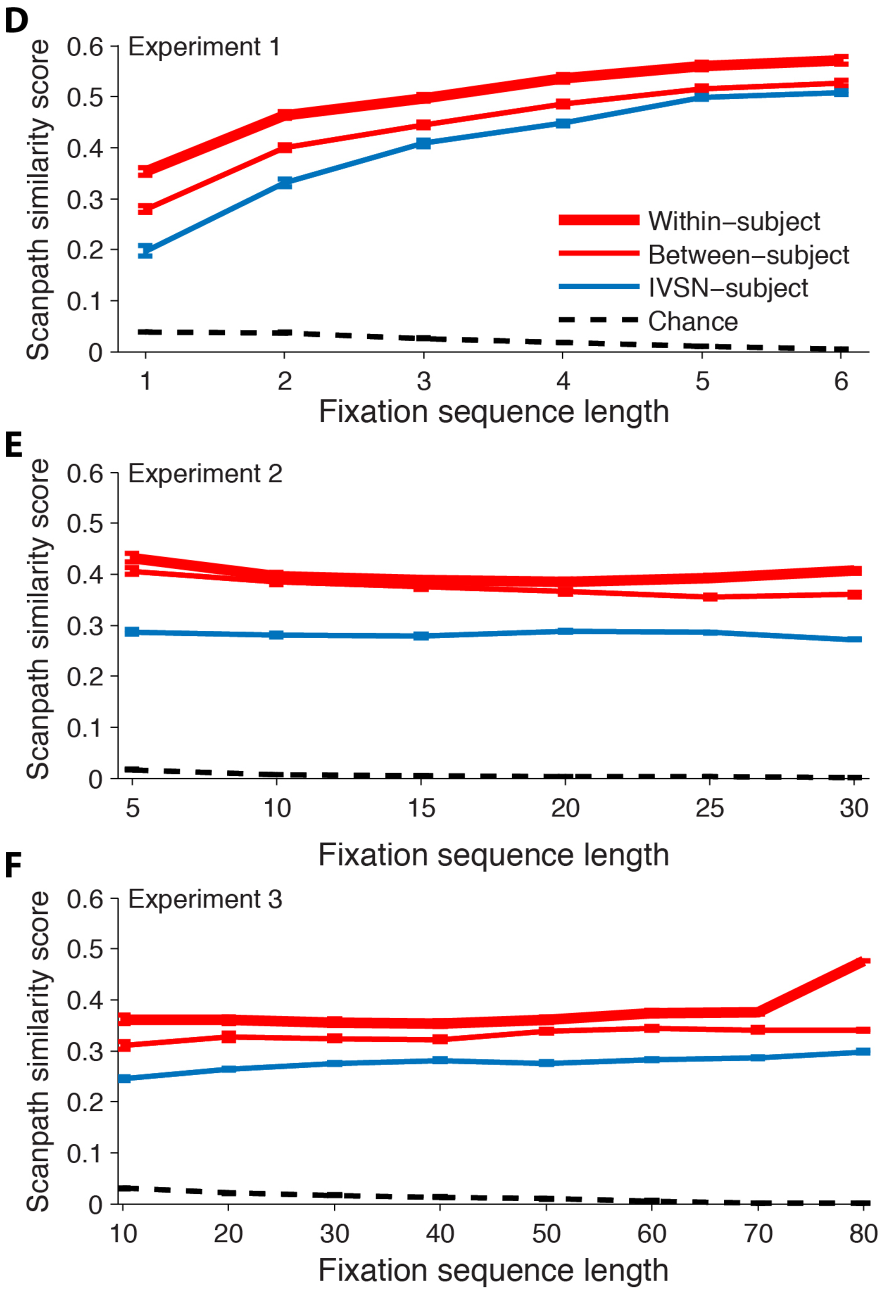

Figure S8: Image-by-image consistency in the spatiotemporal pattern of fixation sequences. Scanpath similarity score (see text and **Methods** for definition) comparing the fixation sequences within subjects (thick red), between-subjects (thin red) and between the IVSN model and subjects (blue) for Experiment 1 (**D**), Experiment 2 (**E**), and Experiment 3 (**F**). The larger the scanpath similarity score, the more similar the fixation sequences are.

In contrast to parts **S8A-B-C**, here only sequences up to a given length were compared. The x-axis indicates the length of sequences compared. For a given fixation sequence length *x*, only sequences of length ≥*x* were considered and only the first *x* fixations were considered. The dashed line indicates the similarity between human sequences and random sequences. Error bars denote SEM, n=15 subjects.

The within-subject similarity score was higher than the between-subject score in all 3 experiments ($p<10^{-9}$). The between-subject similarity score was higher than the IVSN-human score in all 3 experiments ($p<10^{-15}$) and the IVSN-human similarity scores were higher than human-chance scores in all 3 experiments ($p<10^{-15}$).

Supplementary Figure 9

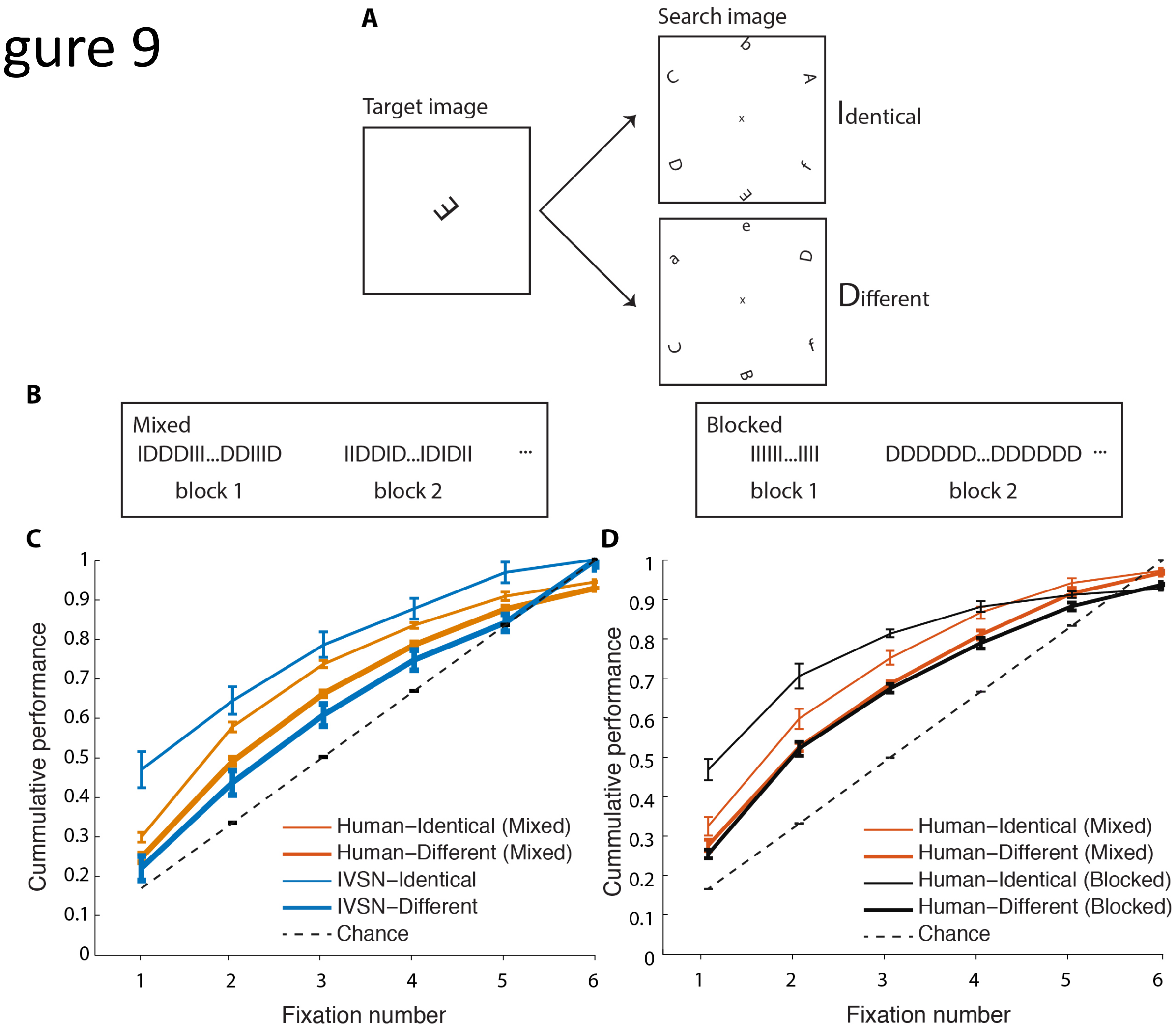

Supplementary Figure 9. Blocked identical trials yielded improved performance (Experiment 1). A. The target as rendered in the target image could be identical (I) to the one in the search image or different (D). **B**. In the mixed condition, all trials were randomized (left). In the blocked condition, all the trials within a block consisted of the target identical condition or the target different condition (right). **C**. In the target identical condition (thin lines), there was an improvement in performance both for humans (red, $p<10^{-7}$, two-tailed t-test, t=5.6, df=4173) and the IVSN model (blue, $p<10^{-5}$, two-tailed t-test t=4.6, df=298) compared to the target different condition (thick lines). **D**. During the experiments reported in the main text, trial order was randomized (Mixed, red). We conducted a separate experiment where trials were blocked such that all Identical trials were together and all Different trials were together (Blocked, black). Within the blocked trials, performance was higher in the Identical trials ($p<10^{-21}$, two-tailed t-test, t=9.8, df=1398). In addition, performance in Identical blocked trials was better than performance in Identical mixed trials ($p<10^{-14}$, two-tailed t-test, t=7.9, df=2236). In contrast, there was no significant difference between the Different blocked trials and the Different mixed trials (p=0.49, two-tailed t-test, t=0.69, df=3335). Error bars denote SEM.

Supplementary Figure 10

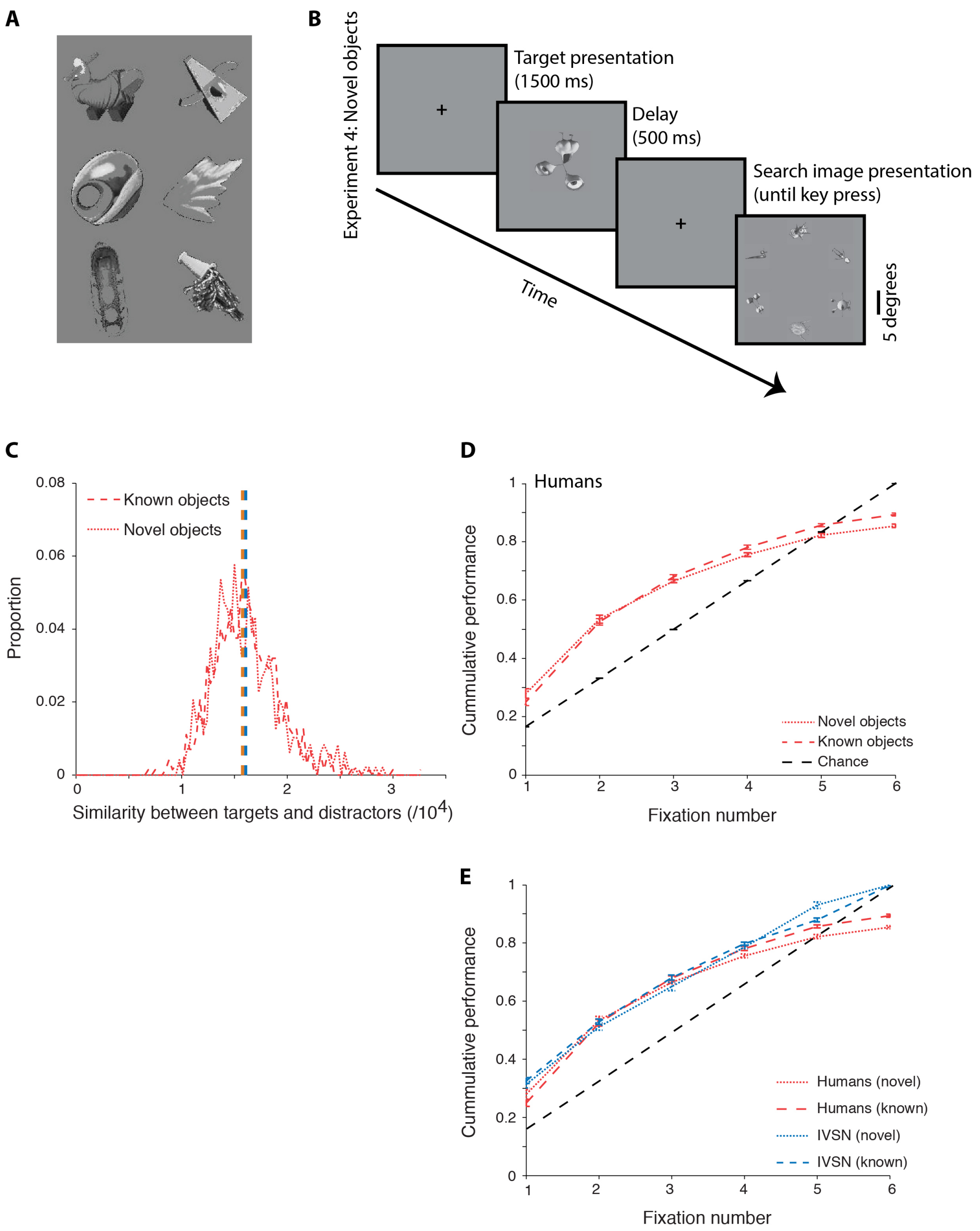

Supplementary Figure 10. Humans can find novel objects.
A. Six example novel objects out of the 1860 novel objects from 98 categories.
B. Schematic of Experiment 4. The novel objects experiment followed the structure of Experiment 1.
C. Difficulty match between known objects (those from Experiment 1) and novel objects. The distribution of similarity scores between targets and distractors for all trials was similar for known objects and novel objects (**Methods**, p>0.6, t=-0.5, df=1204).
D. Cumulative performance following the same format as **Fig. 3E** for known objects (dashed line) and novel objects (dotted line). Performance for both novel and known objects was above chance ($p<10^{-15}$ and $p<10^{-15}$, respectively). There was a small, but significant, difference in performance between novel and known objects (average number of fixations: 2.42+/-1.43 and 2.54+/-1.42, respectively, p=0.004, t=2.9, df=5278, two-tailed t-test). Error bars denote SEM.
E. IVSN model performance for known objects (dashed blue) and novel objects (dotted blue). Human performance is copied from part **D** for comparison. IVSN performance for both novel and known objects was above chance ($p<10^{-17}$ and $p<10^{-12}$, respectively).

The novel objects were collected from the following sources:
1. Horst, J. S., & Hout, M. C. The Novel Object and Unusual Name (NOUN) Database: A collection of novel images for use in experimental research. Behavior Research Methods, 2016. Retrieved from: http://michaelhout.com/?page_id=759.
2. Michael Tarr's web site for Freebles, Greebles, Yadgits, YUFOs: http://wiki.cnbc.cmu.edu/Novel_Objects
3. Alien 3D models: https://www.turbosquid.com/Search/Index.cfm?keyword=alien&max_price=0&min_price=0

Supplementary Figure 11A-C

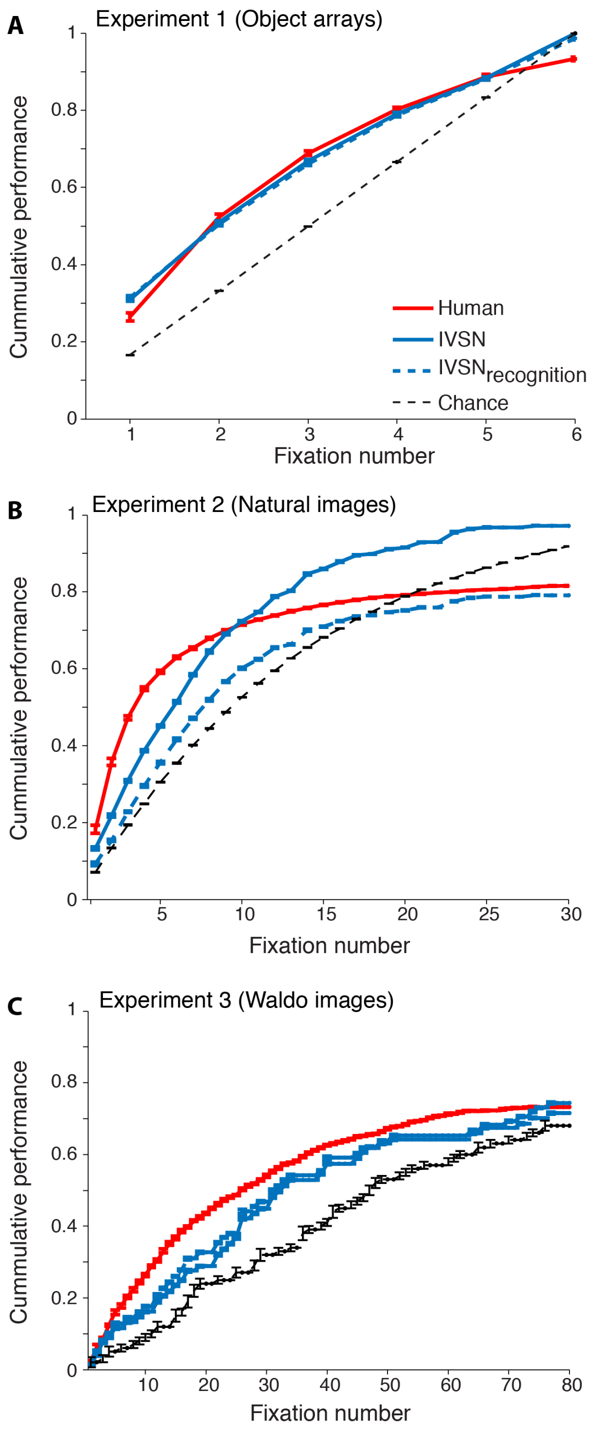

Supplementary Figure 11. Object recognition, memory and saccade sizes. A-C. The results presented in the main text use an "oracle" to determine whether the target is present at a given location or not (**Methods**). Here we introduce a recognition mechanism into the model ($IVSN_{recognition}$) to determine whether the target is present at a given location or whether the model should continue search. These figures match **Figures 3E**, **4E** and **5E** (the red, blue and black dashed lines are copied from those figures for comparison purposes) and introduces the dashed blue line model ($IVSN_{recognition}$). Error bars denote SEM.

The performance of the $IVSN_{recognition}$ model was different from humans in Experiment 2 (two-tailed t-test):
Experiment 1: $p=0.04$
Experiment 2: $p<10^{-5}$
Experiment 3: $p=0.02$

The performance of the $IVSN_{recognition}$ model was significantly better than chance (two-tailed t-test):
Experiment 1: $p<10^{-15}$
Experiment 2: $p<10^{-13}$
Experiment 3: $p<10^{-15}$

Supplementary Figure 11D-F

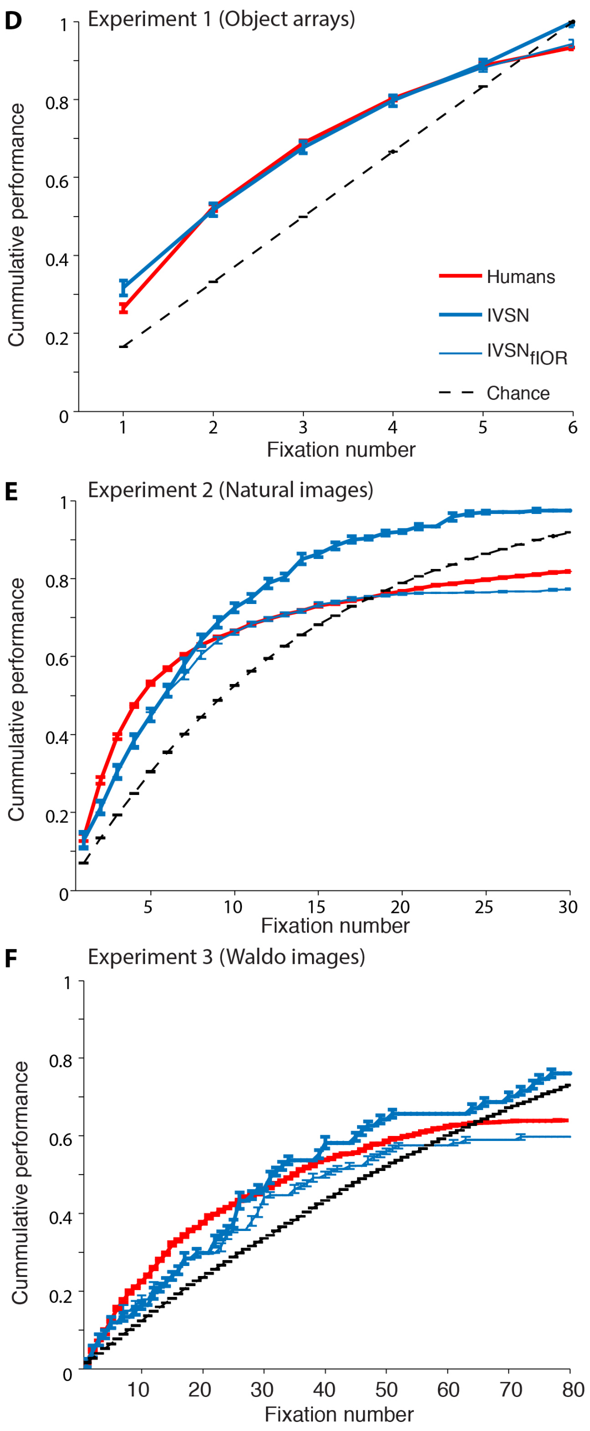

Supplementary Figure 11. Object recognition, memory and saccade sizes. D-F. The model presented in the main text assumes infinite inhibition of return. Here we introduce finite inhibition of return into the model (**Methods**, $IVSN_{fIOR}$. These figures match **Figures 3E**, **4E** and **5E** (the red, blue and black dashed lines are copied from those figures for comparison purposes) and introduces the thin blue line model ($IVSN_{fIOR}$).

The performance of the $IVSN_{fIOR}$ model was not different from humans (two-tailed t-test):
Experiment 1: $p=0.87$
Experiment 2: $p=0.027$
Experiment 3: $p=0.29$

The performance of the $IVSN_{fior}$ model was significantly better than chance (two-tailed t-test):
Experiment 1: $p<10^{-15}$
Experiment 2: $p<10^{-15}$
Experiment 3: $p<10^{-15}$

Supplementary Figure 11G-L

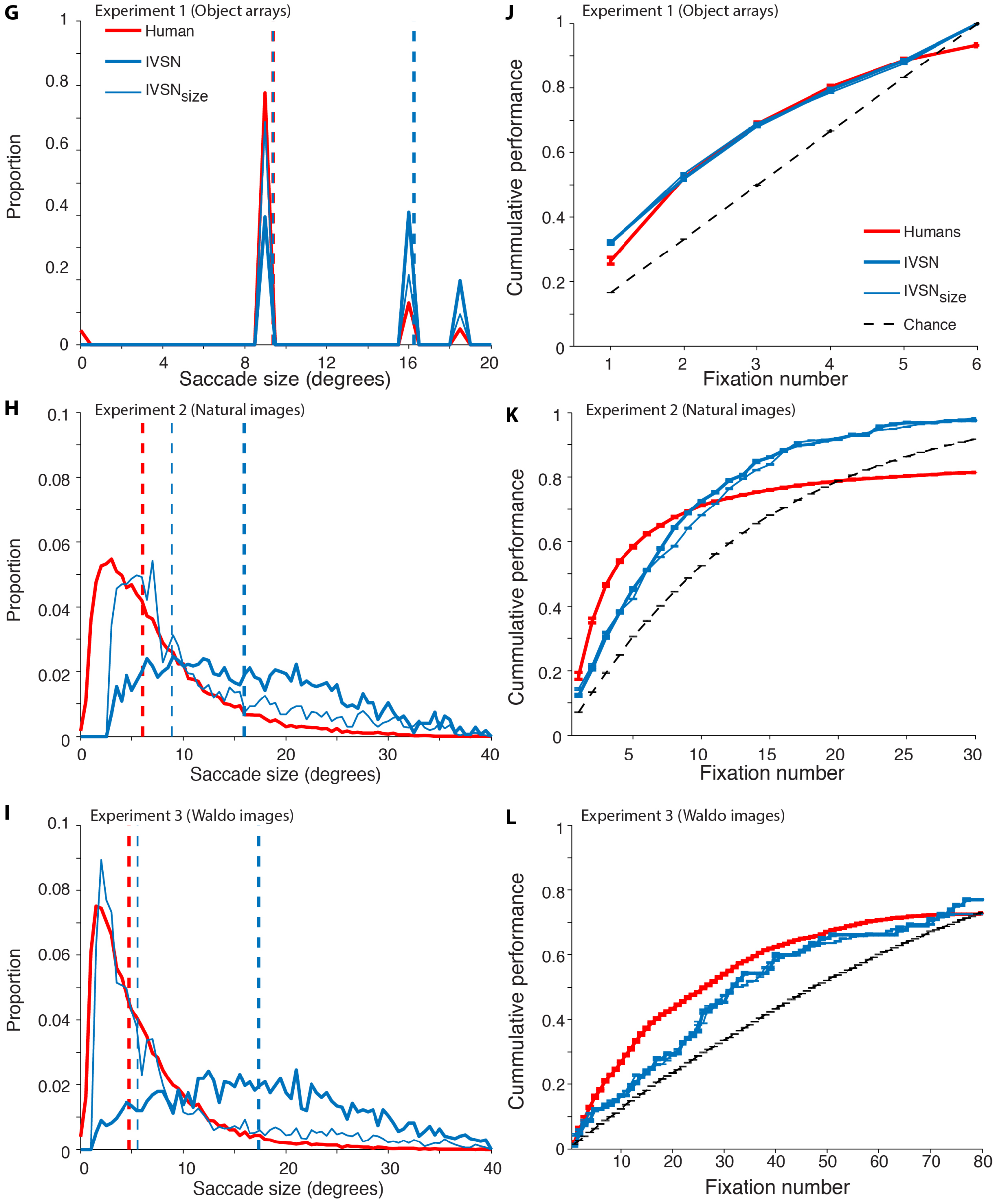

Supplementary Figure 11. Object recognition, memory and saccade sizes. G-I. Distribution of saccade sizes for Experiments 1, 2 and 3, respectively, for humans (red), the IVSN model (thick blue), and the IVSN model constrained by saccade distance ($IVSN_{size}$, thin blue). The vertical dashed lines show the median values. In all experiments, there was a significant difference between humans and the IVSN model ($p<10^{-15}$, two-tailed t-test, t>23).

J-L. Performance of the $IVSN_{size}$ model. The format is the same as that in **Figures 3E**, **4E** and **5E** and the red, thick blue, and black dashed lines are copied from those figures for comparison purposes.

The performance of the IVSNsize model was significantly different from humans (two-tailed t-test):
Experiment 1: p=0.004
Expeirment 2: $p<10^{-12}$
Experiment 3: p=0.002

The performance of the IVSNsize model was significantly different from chance (two-tailed t-test):
Experiment 1: $p<10^{-11}$
Expeirment 2: $p<10^{-14}$
Experiment 3: $p<10^{-15}$

Supplementary Figure 12

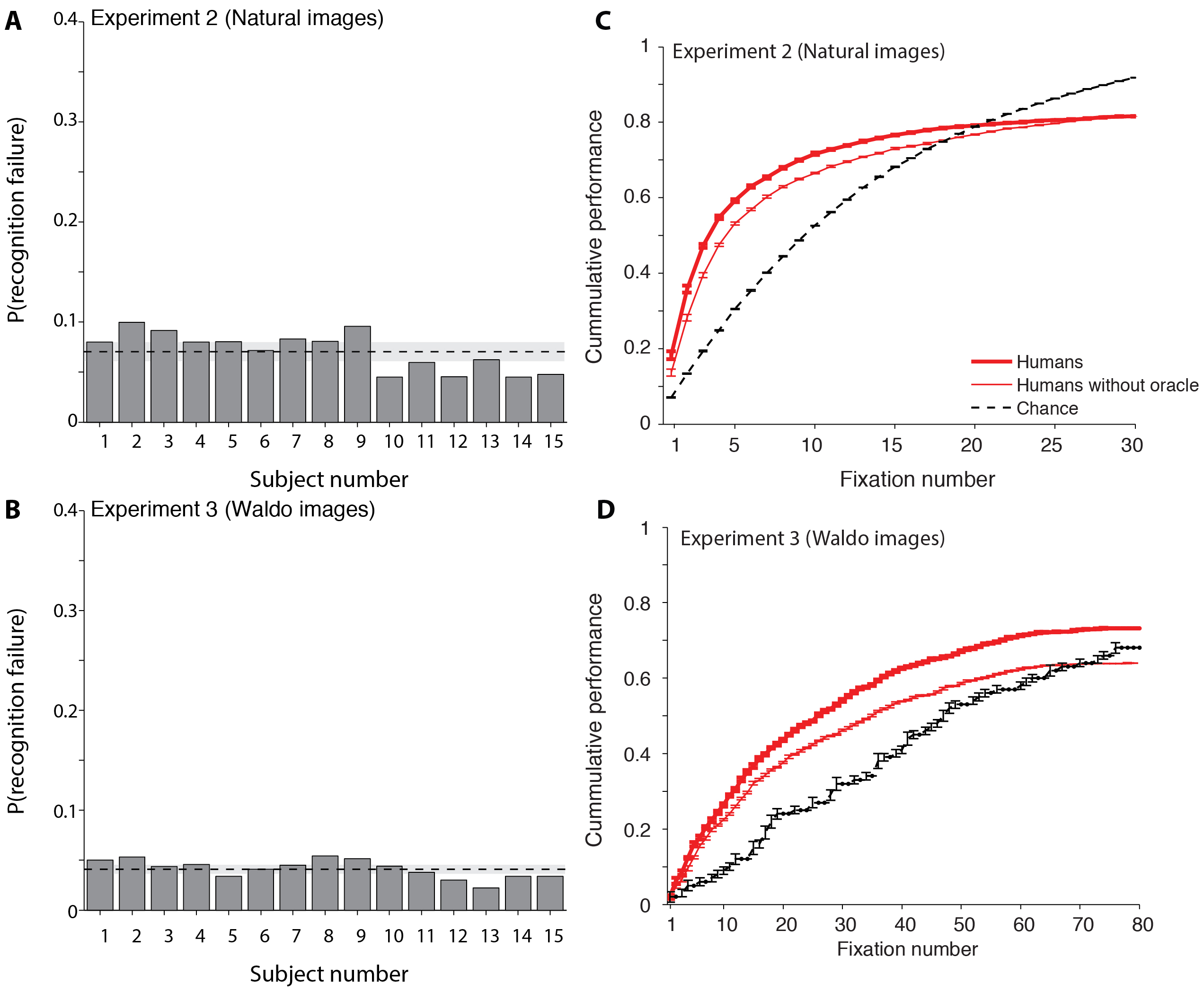

Supplementary Figure 12. Humans may fixate on the target but fail to recognize it. A-B. Probability of fixating on the target and failing to recognize it (not clicking on the target location with the mouse and continuing visual search) for each of the 15 subjects. The dashed line shows the average across subjects; the shaded area is one SD. **C-D**. To directly compare the model and humans, the results presented throughout the text use an oracle to determine whether the target was found or not (except for $IVSN_{recognition}$ in **Figure S11A-C**). If a fixation landed on the target, the target was deemed to be found. In Experiments 2 and 3 -- but not in Experiment 1 -- subjects were asked to indicate the target location with the mouse. Here we compare performance using the oracle version (Humans, thick red line, copied from **Figures 4E** and **5E**) versus performance determined by the time when subjects click the mouse (Humans without oracle, thin red line) for Experiment 2 (**A**) and Experiment 3 (**B**). Human performance without the oracle was also above chance in both cases ($p<10^{-15}$ and $p<10^{-15}$ in **C**, **D**). Human performance with the oracle was different from that without the oracle in Experiment 2 ($p<10^{-15}$, t=11, df=6156), but not in Experiment 3 (p=0.62, t=0.49, df=1375). Error bars denote SEM.

Supplementary Figure 13

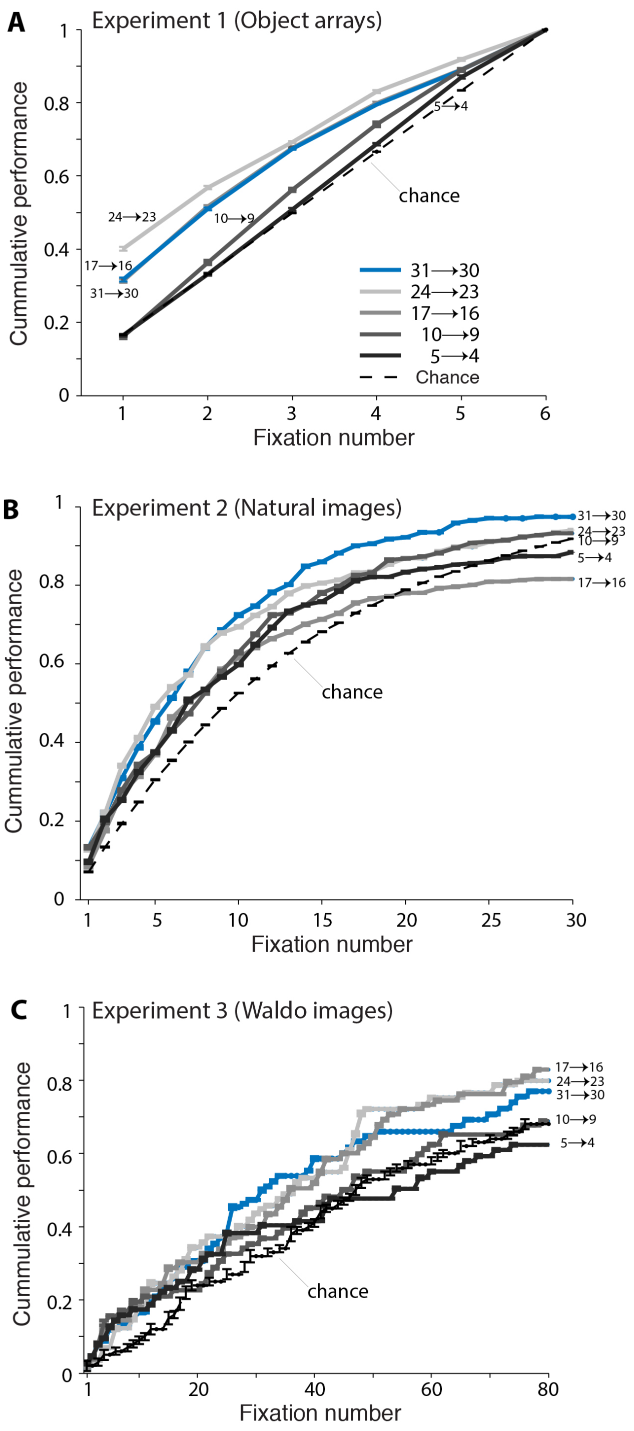

Supplementary Figure 13. Alternative IVSN models with top-down modulation at different levels of the hierarchy. The plots follow the format of **Figures 3E**, **4E** and **5E** and the blue line is copied from those figures for comparison purposes. The curves with different shades of gray show models where top-down modulation is applied at different levels of the ventral stream hierarchy. Error bars denote SEM.

The performance of all models was statistically different from chance (two-tailed t-test, $p<0.01$), except $IVSN_{5\rightarrow4}$ in Experiment 1 (two-tailed t-test, $p=0.39$).

Supplementary Figure 14

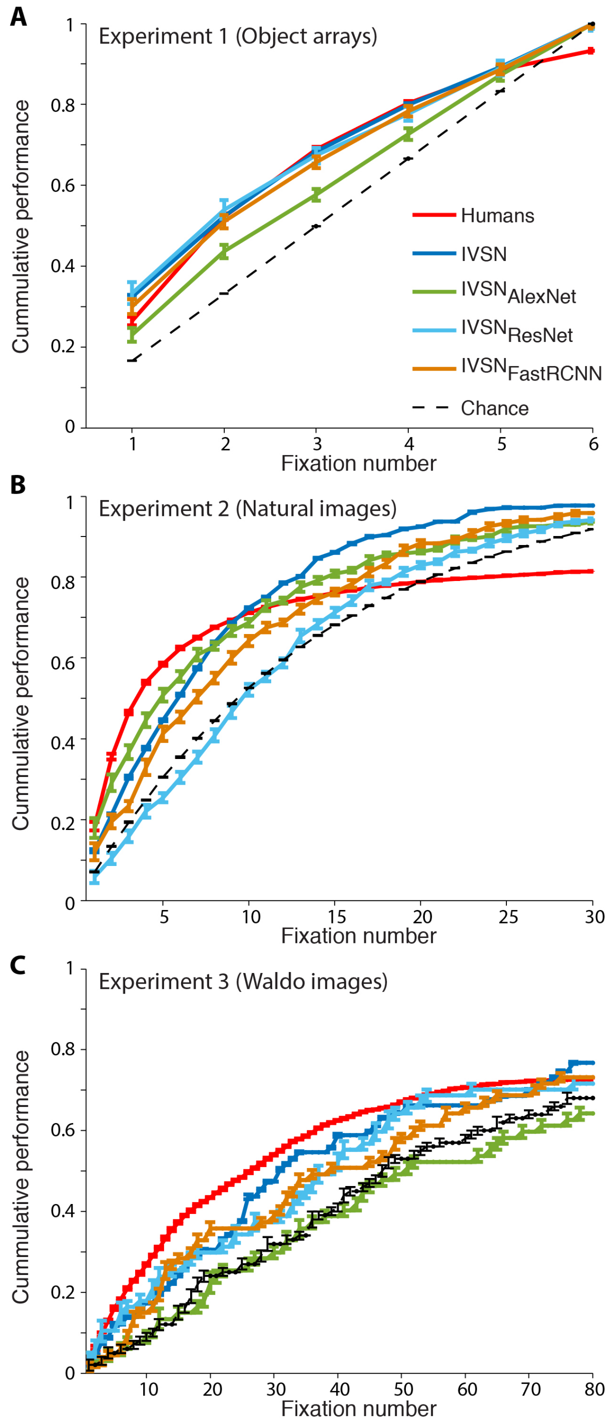

Supplementary Figure 14. Variations on the model with different ventral visual cortex modules show similar performance. The format and conventions for this figure follow those in **Fig. 3E**. The IVSN model performance and chance levels are copied from **Figs. 3E**, **4E** and **5E** for comparison purposes. The other colors denote different models where the ventral visual cortex module in Fig. 2B was replaced by the AlexNet architecture (green), the ResNet architecture (light blue) or the FastRCNN architecture (orange). See Methods for references to these different architectures. The rest of the model remained the same. Error bars denote SEM.

The performance of all models was statistically different from chance (two-tailed t-test, p<0.0006).

4. Supplementary References

1 Borji, A. & Itti, L. State-of-the-art in visual attention modeling. *IEEE transactions on pattern analysis and machine intelligence* **35**, 185-207, doi:10.1109/TPAMI.2012.89 (2013).

2 Krizhevsky, A., Suteskever, I. & Hinton, G. E. Imagenet classification with deep convolutional neural networks. *Advances in Neural Information Processing Systems*, 1097-1105 (2012).

3 He, K., Zhang, X., Ren, S. & Sun, J. in *Proceedings of the IEEE conference on computer vision and pattern recognition.* 770-778 (2016).

4 Ren, S., He, K., Girshick, R. & Sun, J. Faster r-cnn: Towards real-time object detection with region proposal networks. *Advances in Neural Information Processing Systems* 91-99 (2015).

5 Miconi, T., Groomes, L. & Kreiman, G. There's Waldo! A Normalization Model of Visual Search Predicts Single-Trial Human Fixations in an Object Search Task. *Cerebral Cortex* **26**, 3064-3082, doi:10.1093/cercor/bhv129 (2016).

6 Liu, H., Agam, Y., Madsen, J. R. & Kreiman, G. Timing, timing, timing: fast decoding of object information from intracranial field potentials in human visual cortex. *Neuron* **62**, 281-290, doi:10.1016/j.neuron.2009.02.025 (2009).

7 Bichot, N. P., Heard, M. T., DeGennaro, E. M. & Desimone, R. A Source for Feature-Based Attention in the Prefrontal Cortex. *Neuron* **88**, 832-844, doi:10.1016/j.neuron.2015.10.001 (2015).

8 Wolfe, J. M. & Horowitz, T. S. Five factors that guide attention in visual search. *Nature Human Behaviour* **1**, 0058 (2017).

9 Burbank, K., Kreiman, G. in *COSYNE* (2011).

10 Simonyan, K. & Zisserman, A. Very deep convolutional networks for large-scale image recognition. *arXiv*, 1409.1556 (2014).

11 Riesenhuber, M. & Poggio, T. Hierarchical models of object recognition in cortex. *Nature Neuroscience* **2**, 1019-1025, doi:10.1038/14819 (1999).

12 Horowitz, T. S. & Wolfe, J. M. Visual search has no memory. *Nature* **394**, 575 (1998).

13 Klein, R. M. Inhibition of return. *Trends in cognitive sciences* **4**, 138-147 (2000).

14 Schmidhuber, J. & Huber, R. Learning to generate artificial fovea trajectories for target detection. *International Journal of Neural Systems* **2**, 135-141 (1991).

15 Wu, C. C., Wang, H. C. & Pomplun, M. The roles of scene gist and spatial dependency among objects in the semantic guidance of attention in real-world scenes. *Vision Research* **105**, 10-20, doi:10.1016/j.visres.2014.08.019 (2014).

16 Girshick, R., Donahue, J., Darrell, T. & Malik, J. in *Proceedings of the IEEE Conference on Computer Vision and Pattern Recognition* 580-587 (2014).

17 Yang, J. & Yang, M. H. Top-down visual saliency via joint crf and dictionary learning. *Computer Vision and Pattern Reconigition*, 2296-2303 (2012).

18 Perronnin, F. & Larlus, D. in *Proceedings of the IEEE conference on computer vision and pattern recognition.* 3743-3752 (2015).

19 Gevers, T. & Smeulders, A. W. PicToSeek: combining color and shape invariant features for image retrieval. *IEEE transactions on image processing : a publication of the IEEE Signal Processing Society* **9**, 102-119, doi:10.1109/83.817602 (2000).
